# Time-resolved proteomic profiling of *Cupriavidus metallidurans* CH34 in the copper-induced viable-but-nonculturable state

**DOI:** 10.1093/mtomcs/mfaf007

**Published:** 2025-02-17

**Authors:** Timothej Patocka, Surya Gupta, Felice Mastroleo, Natalie Leys, Jean-Yves Matroule, Rob Van Houdt

**Affiliations:** Microbiology Unit, Nuclear Medical Applications, Belgian Nuclear Research Centre, SCK CEN, Mol, Belgium; Research Unit in Biology of Microorganisms, University of Namur, Namur, Belgium; Microbiology Unit, Nuclear Medical Applications, Belgian Nuclear Research Centre, SCK CEN, Mol, Belgium; Microbiology Unit, Nuclear Medical Applications, Belgian Nuclear Research Centre, SCK CEN, Mol, Belgium; Microbiology Unit, Nuclear Medical Applications, Belgian Nuclear Research Centre, SCK CEN, Mol, Belgium; Research Unit in Biology of Microorganisms, University of Namur, Namur, Belgium; Microbiology Unit, Nuclear Medical Applications, Belgian Nuclear Research Centre, SCK CEN, Mol, Belgium

## Abstract

Copper-based materials are actively explored for their potential as antimicrobial agents. However, recent studies show that sublethal concentrations of Cu ions can induce the viable-but-nonculturable (VBNC) cell state in certain bacteria, hampering contamination control, and monitoring. In this study we contribute to the unravelling of this largely enigmatic phenomenon by determining the time-resolved proteome of Cu-treated *Cupriavidus metallidurans* CH34 during VBNC induction and resuscitation. High-throughput quantitative liquid chromatography tandem mass spectrometry (LC-MS/MS) analysis was performed at multiple sample time points, revealing the cellular adaptations that trigger VBNC formation and the characteristic spontaneous recovery of culturability. Entry into the VBNC state correlated with a widespread response to oxidative stress as well as downregulated pyruvate metabolism. The expression of specific metal resistance determinants changed with Cu exposure time and culminated in the strong upregulation of proteins linked to periplasmic Cu ion detoxification during the resuscitation phase. We suggest that this delayed induction of Cu resistance proteins is paralleled by the gradual reconstitution of energy reserves through metabolization of intracellular polyhydroxybutyrate, as supported by flow cytometric fluorescence measurements. Furthermore, Cu-treated cells showed upregulation of several motility and chemotaxis proteins, and increased cell motility was observed phenotypically. Our results reveal a highly dynamic proteomic response, provide fundamental insights into the VBNC state and emphasize the advantages of time-resolved proteomic analysis.

## Graphical Abstract

**Graphical Abstract:**
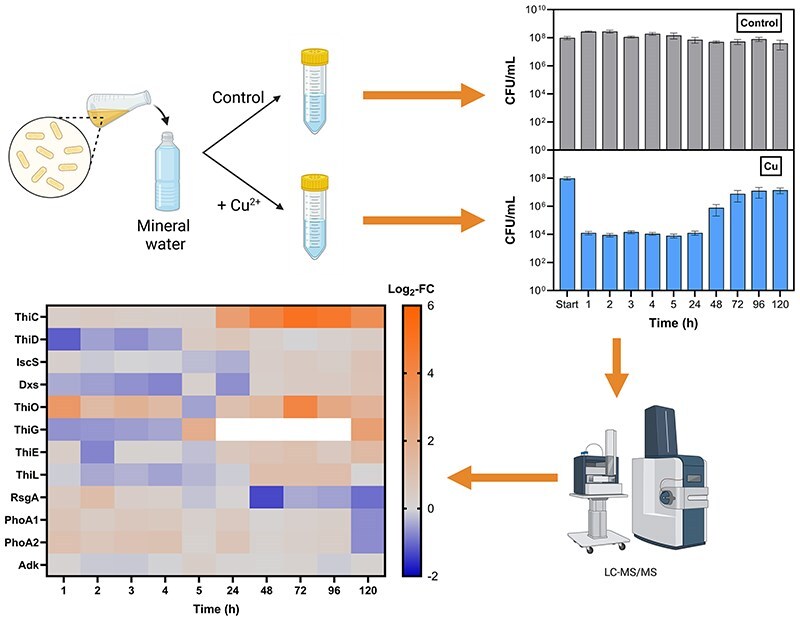

## Introduction

The use of copper-based antimicrobials is a re-emerging strategy for decontamination purposes in agriculture, food industry, health settings, and even spaceflight applications [1–5]. Before implementation, the bacteriostatic effect of Cu ions needs to be thoroughly tested. It has been shown that sublethal concentrations of Cu ions can induce the so-called viable-but-nonculturable (VBNC) cell state in several bacterial species [6]. This VBNC state is thought to be a cellular survival strategy with the purpose of ensuring viability under harsh environmental conditions such as nutrient depletion, drastic temperature shifts, or exposure to metals, among others [7]. A central feature of VBNC cells is their inability to grow on regular microbiological media. This is a concern when relying on culture-based testing for bacterial diagnostics and monitoring as it could lead to false negative results [8]. Under more favorable conditions, VBNC cells can restore culturability, a phenomenon called resuscitation. During resuscitation, vital cellular functions are recovered, including the virulence of pathogenic bacteria that might pose a risk in settings such as public health [9, 10].

Resuscitation is usually (but not necessarily) triggered by external factors reverting the unfavorable conditions, such as nutrient supplementation, metal ion chelation, but also biological stimulation [11, 12]. Conversely, spontaneous resuscitation based on the activation of intrinsic factors is rarely described. Recently, Maertens *et al*. found that water-borne cells of *Cupriavidus metallidurans* enter the VBNC state upon exposure to low levels of Cu ions, and spontaneously resuscitate after 7 days of incubation [13]. *Cupriavidus metallidurans*, a soil bacterium from the *Burkholderiaceae* family, is a model organism for the study of bacterial metal resistance. Type strain CH34 features a remarkable amount of metal resistance genes, many of which are located on one of its two megaplasmids [14, 15]. In the aforementioned study, resuscitation was only possible when these plasmid-encoded metal resistance determinants were present [13]. This finding highlighted an interesting association between the VBNC state and bacterial Cu resistance. In the present study, we are further investigating this association at a proteomic level. In recent years, efforts to elucidate cellular mechanisms controlling the VBNC state have increasingly focused on the application of omics techniques [11]. However, only a few studies explored the Cu-induced VBNC state [6]. Here, we analyzed whole proteome adaptations of *C. metallidurans* CH34 during a Cu-induced VBNC state and, notably, during spontaneous resuscitation. Rather than comparing only two different cell populations (e.g. before and after VBNC induction), our aim was to capture time-resolved proteomic changes through sampling at multiple time points. This approach allowed us to gain valuable insights into the dynamics of the VBNC state in *C. metallidurans* CH34, serving as a stepping stone toward the unravelling of the enigmatic VBNC state which can eventually help to improve countermeasures against bacterial contaminations.

## Material and methods

### Bacterial strains and culture conditions

*Cupriavidus metallidurans* strain CH34 was routinely grown in Tris-buffered minimal medium (MM284) [16] with 0.2% Na-gluconate (VWR chemicals, Leuven, Belgium) at 30°C. Liquid cultures were grown in a shaking incubator at 180 rpm. Agar plates were prepared by adding 2% bacteriological agar (Oxoid, Hampshire, UK). For motility assays, 0.4% agar plates were prepared.

### VBNC induction, resuscitation, and motility assays

Fully grown cultures were washed twice with filter-sterilized (0.2 µm filter) bottled mineral water (Ordal, Belgium and Evian, France; additional information on their composition can be found in Table S1) and diluted to a starting inoculum of 10^8^ cells/ml in the same mineral water. To induce the VBNC state, 10 µM CuSO_4_ (Merck KGaA, Darmstadt, Germany) was added, while no CuSO_4_ was added to the control condition, and samples were incubated in a shaking incubator at 30°C. Where applicable, 5.6 mM thiamine hydrochloride (Sigma-Aldrich, Saint Louis, Missouri, USA) was added. Samples were drawn after 0, 1, 2, 3, 4, 5, 24, 48, 72, 96, and 120 h of incubation, and the total viable count was determined by plating 100 µl of a serial 10-fold dilution (in sterile PBS) on MM284 agar and counting after 3 days at 30°C. To test swarming potential, 10 µl of cell suspension from samples at time points 48, 72, 96, and 120 h was plated onto MM284-gluconate plates containing 0.4% bacterial agar. After 3 days of incubation at 30°C, the visible swarming diameter was measured with a ruler and divided by corresponding CFU counts to yield the normalized swarming range. All experimental conditions were carried out in biological triplicates. Statistical analysis was performed using unpaired *t*-tests implemented in GraphPad Prism 10.

### Cell counting, viability determination, and PHB quantification via flow cytometry

All flow cytometry assays were performed on a NovoCyte Quanteon flow cytometer (Agilent, Santa Clara, California, USA).

Similar to the viable count, samples were drawn after 0, 1, 2, 3, 4, 5, 24, 48, 72, 96, and 120 h of incubation. Viability was assessed with a SYBR Green/PI assay. Briefly, cell suspensions were 100-fold diluted in filtered mineral water and SYBR Green (Life technologies, Carlsbad, California, USA) and propidium iodide (Merck KGaA, Darmstadt, Germany) were added to a final concentration of 1X (starting from a 10 000X commercial stock solution) and 200 µM, respectively. Suspensions were incubated in the dark at 37°C for 20 min to allow complete binding of the dyes. Stained cells (including autoclaved cells as a control for dead cells) were analyzed in “fast” flow mode and fluorescence was acquired using the FITC and PI detection channels. Additionally, total cell numbers in unstained cell suspensions were enumerated based on manual forward vs side scatter (FSC vs SSC) gating, excluding background events.

To quantify intracellular polyhydroxybutyrate (PHB) levels, the protocol described in [17] was adapted to flow cytometry: Nile Red stock solution (1 mg/ml) was prepared by dissolving Nile Red solid dye (Sigma-Aldrich, Saint Louis, Missouri, USA) in dimethyl sulfoxide (DMSO; Sigma-Aldrich, Saint Louis, Missouri, USA) and filter-sterilizing the solution through a 0.45 µm DMSO-proof filter. *C. metallidurans* cells were diluted to approximately 10^6^ cells/ml in filtered mineral water and stained by adding Nile Red stock solution to a final dye concentration of 1 µg/ml, followed by a 30-min incubation step at 30°C. Stained and unstained samples (including a water blank) were analyzed in “fast” flow mode and fluorescence was acquired using the PE-Cy5 detection channel.

Data analysis was carried out using the provided NovoExpress software (Agilent, Santa Clara, California, USA) and GraphPad Prism 10. For PHB quantification, median fluorescence intensities were compared using Mood's median test and effect sizes were calculated using Cramér's V [18].

### Protein extraction

Samples for protein extraction were taken every 60 min until 5 h of incubation, as well as 24, 48, 72, 96, and 120 h after the start of the experiment. Samples were pelleted and washed twice with ice-cold PBS (Life technologies, Carlsbad, California, USA). Pellets were then resuspended in lysis buffer [2% sodium dodecyl sulfate (Life technologies, Carlsbad, California, USA) in 50 mM ammonium bicarbonate aqueous solution (Sigma-Aldrich, Santa Clara, California, USA)], vortexed for 30 s and subsequently incubated at 95°C for 5 minutes. After cooling on ice, lysis was completed through sonication using a UP50H ultrasonic processor (Hielscher, Teltow, Germany). The lysates were centrifuged at 14 000 g and 4°C for 20 min and supernatants were stored at −80°C for further processing. Protein concentrations in the crude extracts were determined using the bicinchoninic acid kit for protein determination (Sigma-Aldrich, Santa Clara, California, USA) following the manufacturer's instructions.

### Protein sample processing

Extracted proteins were further processed using the suspension trapping method, S-trap (Bioconnect, Huissen, The Netherlands), following the manufacturer's instructions.

The LC-MS/MS analysis was performed using a nanoElute UHPLC (Bruker Daltonics, Bremen, Germany) connected to a QTOF-MS instrument (Impact II, Bruker Daltonics, Germany) via a CaptiveSpray nanoflow electrospray source (Bruker Daltonics, Bremen, Germany). Samples were run in a random order in order to identify a potential batch effect. In total, 2 µg of tryptic digest (in solvent A) was injected onto a trapping column setup (300 µm x 5 mm, C18 PepMap 300, 5 µm, 100 Å; Bruker Daltonics, Bremen, Germany). Subsequently, peptides were separated using a C18 Reprosil AQ, 1.9 µm, 120 Å, 0.075 × 150 mm column operated at 40°C (Bruker Daltonics, Bremen, Germany) at a flow rate of 0.2 µl/min. Gradient conditions were: 2%–35% solvent B for 100 min; 35%–95% solvent B for 10 min; and 95% solvent B held for 10 min (solvent A, 0.1% formic acid in water; solvent B, 0.1% formic acid in acetonitrile). Drying gas flow and temperature of the CaptiveSpray were set to 4 l/min and 180°C, respectively, and nebulizer gas pressure was set to 0.4 bar. The MS acquisition rate was set to 2 Hz and data have been acquired over a 150–2200 m/z mass range. In all the full-scan measurements, a lock-mass (m/z 1221.9906, Hexakis (1H, 1H, 4H-hexafluorobutyloxy)phosphazine) (Bruker Daltonics, Bremen, Germany) was used as an internal calibrator.

The Instant Expertise method (Compass otofSeries 4.1, Bruker Daltonics, Bremen, Germany) was used to select as many as possible of the most intense ions per cycle of 3 s MS/MS accumulation depending on the MS1 level. The threshold (per 1000 summation) absolute was 2500 cts (spectral rate of 2 Hz). Peptide fragmentation was performed with nitrogen gas on the most abundant and at least doubly charged to five charged ions detected in the initial MS scan. Active exclusion was performed after 1 spectrum for 0.50 min unless the intensity of the precursor ions was more than 3 times higher than in the previous scan.

### Proteomics data analysis

All raw mass spectrometry spectra files were processed using MaxQuant software version 2.0.1.0 and proteins were identified with the built-in Andromeda search engine [19, 20]. In total, 60 raw files, with three replicates of each 20 conditions (control and Cu-induced at 10 different time points) were processed in parallel. The MS/MS spectra searches were performed with a database containing all *C. metallidurans* CH34 (Taxonomy ID: 266 264) UniProt protein sequences (downloaded from *ftp.uniprot.org* on 2022-03-30). Searches were performed with default MaxQuant parameter settings with cysteine carbamidomethylation as fixed modification, and methionine oxidation and Protein N-terminal acetylation as variable modification. False-discovery rate cutoffs were set to 1% on peptide, protein, and site decoy level, trypsin as a digestion enzyme and seven amino acids as minimum peptide length. The resulting data from MaxQuant with minimum two unique peptides was retained, processed to remove reverse hits and contaminants, and Log_2_ transformed for further analysis. The technical variation among replicates was removed by median normalization on Log_2_-transformed intensities. Missing values in the data were replaced by values from a normal distribution with settings width = 0.3 and downshift = 1.8 [21]. Sample distribution was examined via density and box plots, as well as PCA analysis (Figs S1–S3). Protein differential expression was calculated using the LIMMA R package [22], based on the empirical Bayes moderated test-statistics. Differential expression was considered significant at a *q*-value < 0.05 and FC ≤ 0.67 or ≥ 1.5 (|Log_2_-FC| ≥ 0.5849).

## Results

### *Cupriavidus metallidurans* enters the VBNC state upon Cu stress and resuscitates spontaneously

**Figure 1.**
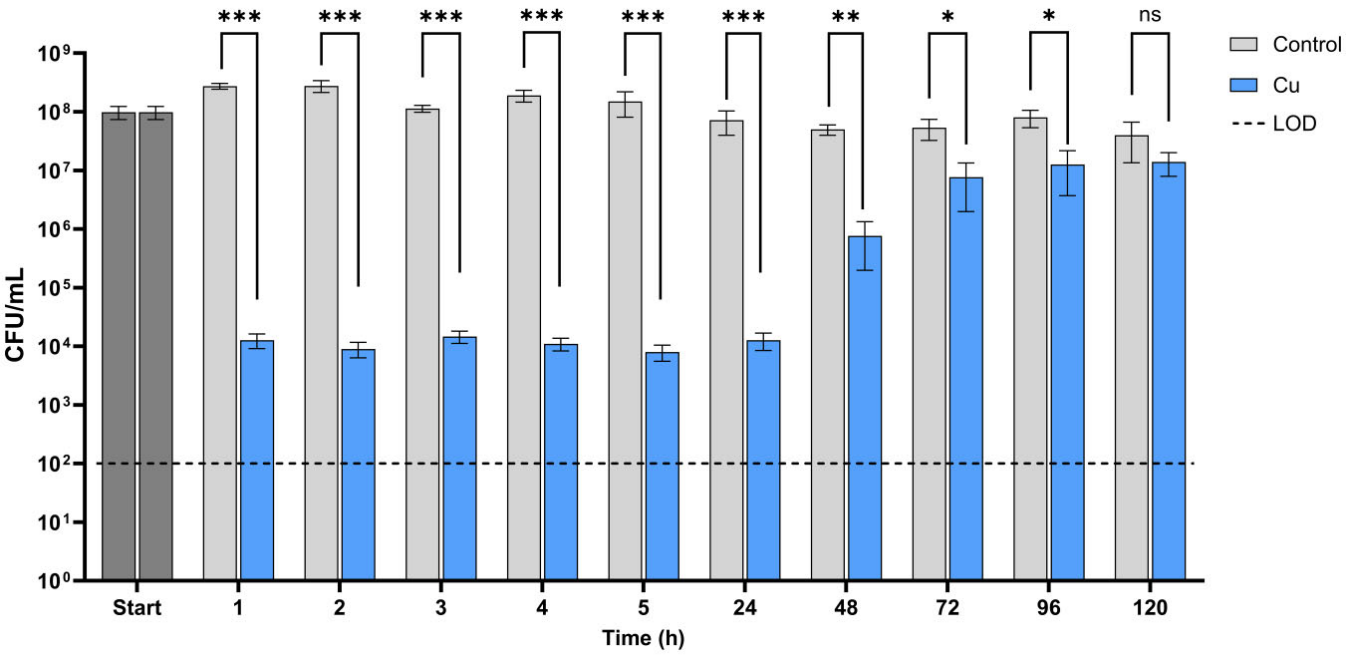
*Cupriavidus metallidurans* CH34 was incubated in mineral water ± 10 µM CuSO_4_ leading to loss of culturability in Cu-treated cells after 1 h and gradual restoration until complete resuscitation at 120 h. Significant difference: *P < .05, **P < .01, ***P < .001, ns = no significant difference. LOD = limit of detection.

Maertens *et al*. identified the transition into a VBNC state when *C. metallidurans* cells are exposed to elevated Cu^2+^ levels in drinking water, showing spontaneous resuscitation within several days of incubation [13]. In a first step, we corroborated this observation and added sampling time points as well as statistical analyses. Mineral water (Ordal) was inoculated with cells of type strain CH34 to a starting cell number of approximately 10^8^ cells/ml and supplemented with 10 µM CuSO_4_ (no CuSO_4_ in control condition). Colony forming units were assessed during the first 5 h and every 24 h after starting the experiment (Fig. 1). In agreement with Maertens *et al*., the number of culturable cells decreased by 4 Log during the first hours of Cu treatment. Already after 1 h, Cu-treated cells showed a significant reduction in culturability (*P* < .001). From 24 h (1 day) after the start of Cu treatment, a gradual increase in CFU counts could be observed, while cell numbers in the control condition were staying relatively constant. Until 96 h (4 days), the reduction in culturability was still significant (*P* < .05). From 120 h (5 days) onwards, no statistical difference between Cu and control condition could be observed, corresponding to the complete resuscitation (Fig. 1). Throughout the experiment, the total cell numbers as assessed by flow cytometry were not significantly different between the Cu-treated and control conditions (Fig. S4). In addition, cell viability was also assessed with a SYBR Green/PI assay that monitors viability based on membrane integrity, which indicated that 10%–20% of Cu-treated cells remained viable between 24 and 120 h of exposure (Fig. S4). Finally, to verify that VBNC induction and resuscitation was not unique to the used mineral water, we tested an additional mineral water (Evian; Fig. S5), which showed the same phenomena.

### VBNC and resuscitating cells show dynamic proteomes

**Figure 2.**
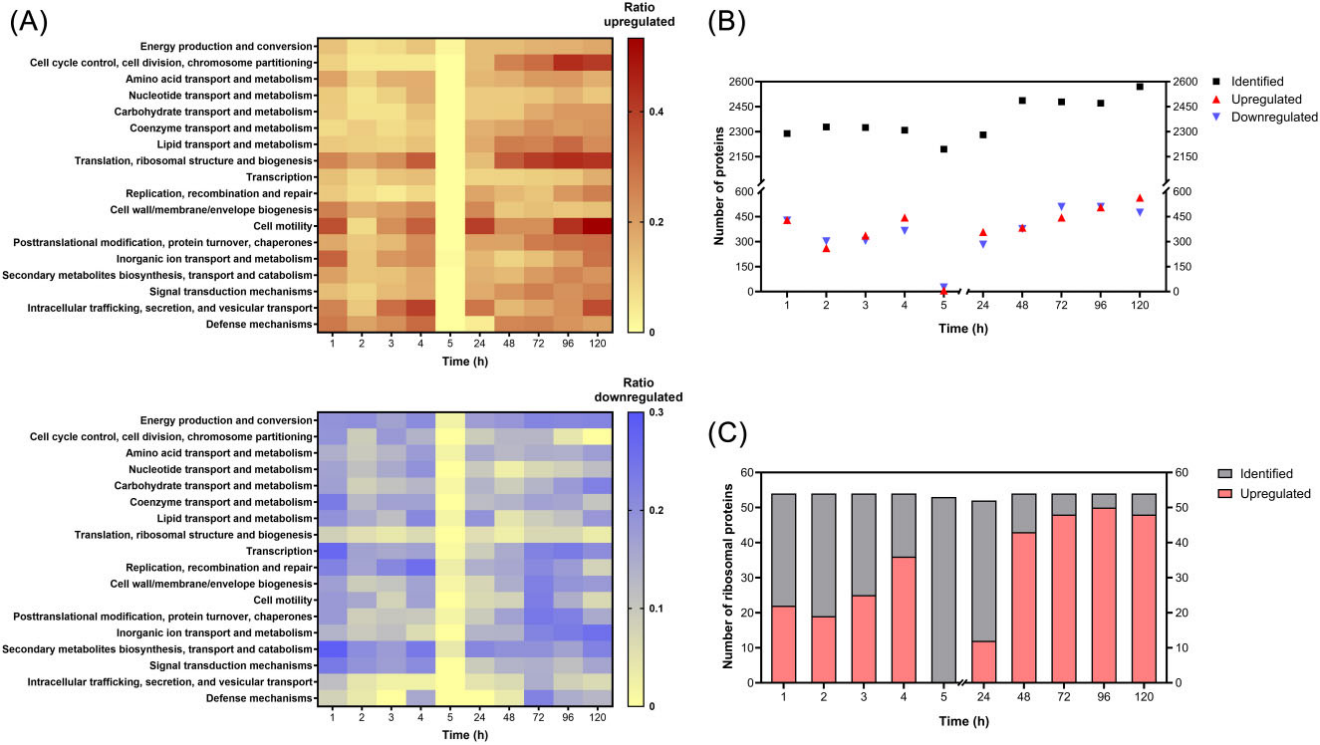
(A) COG analysis: ratio of up/downregulated among identified proteins for different COG classes at different time points. Excluded: time point 5 h, “Function unknown” class, uncategorized proteins. (B) Number of identified and up/downregulated proteins at each time point. (C) The number of identified and upregulated ribosomal proteins (RPs) for each sample time point.

To investigate the proteomic state of VBNC cells and during resuscitation, cells were pelleted for protein extraction at the following sample time points: 1, 2, 3, 4, 5, 24, 48, 72, 96, and 120 h of incubation (Cu and control condition). Proteome analyses were performed via protein extraction and subsequent LC-MS/MS analysis. All detected proteins, including data from single replicates, are listed in Table S2. The number of identified proteins for each time point ranged between 2195 and 2570 (Table 1, Fig. 2B). In general, a good coverage of CH34’s annotated proteome could be reached, with an average of 2373 identified proteins, which amounts to 36.6% of the 6488 annotated coding sequences in the Kyoto Encyclopedia of Genes and Genomes (KEGG) database [23]. For each time point, DE proteins were determined by comparing their abundance in Cu-treated and control samples. Proteins with a FC ≤0.67 or ≥1.5 (|Log2-FC| ≥0.5849) and *q*-values <0.05 were considered DE (Table 1). The number of DE proteins was the lowest at 5 h with 32 DE proteins (Fig. 2B). Afterward, the number of DE proteins gradually increased to reach a maximum at 120 h (1038 DE proteins).

**Table 1.** Number of identified and differentially expressed (DE) proteins at each sample time point

	Time (h)									
	1	2	3	4	5	24	48	72	96	120
Identified	2289	2328	2325	2309	2195	2281	2487	2479	2471	2570
Upregulated	430	261	337	445	7	358	384	445	506	564
Downregulated	427	302	305	365	25	282	376	509	509	474
DE	857	563	642	810	32	640	760	954	1015	1038

Identified proteins were categorized based on Clusters of Orthologous Genes (COGs) [24]. For each COG class, the number of up-/downregulated proteins among the total number of proteins linked to the COG class was determined and represented as a ratio. Repeating this step for every time point, we obtained an overview of proteomic adaptations in VBNC and resuscitating cells (Fig. 2A). At most time points, high numbers of upregulated proteins (ratios up to 0.5) were observed in the classes “Translation, ribosomal structure and biogenesis,” “Cell motility,” and “Intracellular trafficking, secretion, and vesicular transport.” These classes coincidently showed low numbers of downregulated proteins (ratios partly below 0.1). In contrast, many proteins were continuously downregulated in e.g. “Energy production and conversion,” “Transcription,” “Replication, recombination, and repair” and “Secondary metabolites' biosynthesis, transport and catabolism.” For other classes, the ratios fluctuated more strongly at different phases of incubation. “Cell cycle control, cell division, chromosome partitioning” as well as “Lipid transport and metabolism” (among others) showed high numbers of upregulated proteins between 48 and 120 h (2–5 days, resuscitation phase), but not in the early incubation stage (1–4 h, VBNC induction phase). Conversely, the class “Cell wall/membrane/envelope biogenesis” had many upregulated proteins only in the early incubation stage. Based on COG analysis, we can assume that cells going through the VBNC state and resuscitation are characterized by highly dynamic proteomic profiles. Next, we showcased in detail the similarities and differences between the proteomes of these two cell states.

### Motility is activated by Cu treatment

**Figure 3.**
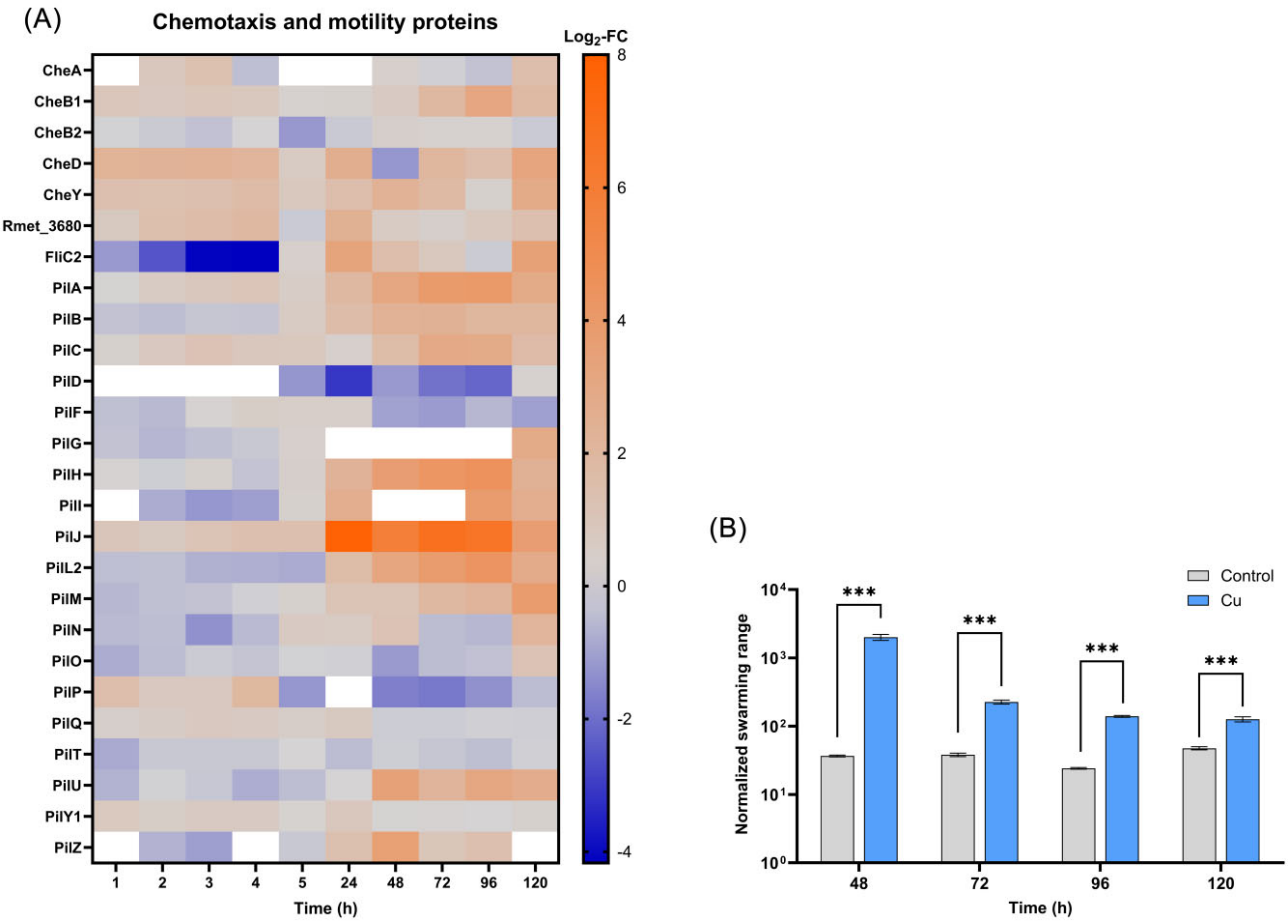
(A) Log_2_-fold change (Log_2_-FC) values of chemotaxis and motility proteins (Cu-treated vs control condition) at each time point. white = not identified. (B) The normalized swarming range of Cu-treated cells was increased compared to control cells. ***Significant (*P* < .001) difference.

A noticeable feature of Cu-treated CH34 cells was the strong expression of proteins linked to cell motility, regardless of incubation time (Fig. 3A). While the chemotaxis protein CheA was upregulated only at day 5, CheB1, CheD, and CheY were upregulated at almost all time points. The CheY-like response regulator Rmet_3680 was also found upregulated at several time points (2–4 h, 24 h, and 5 days). Interestingly, the flagellin protein FliC2 was downregulated in the first 4 h of incubation, but strongly upregulated in later time points, showing Log_2_-FC value of 3.25 (24 h) and 3.48 (5 days). During the VBNC phase, several negative but nonsignificant Log_2_-FC values were obtained for certain proteins of the Pil family (e.g. PilL2, PilM, PilN, PilO). In general, however, the data points toward higher abundance of Pil proteins in Cu-treated samples, especially during the resuscitation phase (e.g. PilJ: Log_2_-FC > 5.0 for days 1–4).

To evaluate whether this proteomic response translated to a phenotypic change, we tested cellular motility by plating cell suspensions on 0.4% agar plates. The swarming diameter after a 3-day incubation period was measured for time points 48, 72, 96, and 120 h and normalized by the corresponding CFU counts. Samples from time points before 48 h were not included in this assay since an accurate measurement of the swarming range was impeded by the lack of culturable cells. The average swarming range was significantly higher (*P* <0.001) in all Cu-treated samples compared to the control condition (Fig. 3B). This result complies with our proteomics data, indicating increased motility in cells experiencing and resuscitating from a Cu-induced VBNC state.

### Ribosomal proteins are upregulated throughout Cu exposure

Proteomic profiling revealed that many proteins linked to translation were upregulated in both VBNC and resuscitated cells (Fig. 2A). In fact, a strikingly high number of significantly upregulated ribosomal proteins (RPs) was found at most time points (Fig. 2C). Remarkably, after 1 h of Cu-treatment, already 41% (22 out of 54) of the identified RPs were upregulated. Strikingly, at 5 h, none of the 53 identified RPs were DE. However, 23% (12 out of 52) of the identified RPs were again upregulated at 24 h, which increased to a maximum of 93% (50 out of 54) at 96 h. Between days 2 and 5 of incubation (48–120 h), at least 80% of the identified RPs were upregulated, suggesting a need for enhanced translation and/or the preparation for cell division during the resuscitation phase.

### Cell division proteins are upregulated during resuscitation

**Figure 4.**
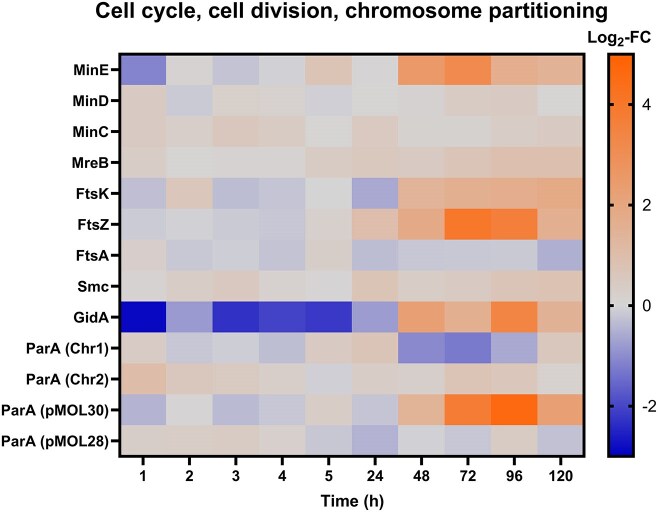
Log_2_-fold change (Log_2_-FC) values of several proteins linked to COG class “Cell cycle, cell division, chromosome partitioning” (Cu-treated vs control condition) at each time point. Chr1 = chromosome, Chr2 = chromid.

Several proteins linked to cell division were upregulated in Cu-treated cells between days 2 and 5 (48–120 h, Fig. 4) during which resuscitation took place. The cell division topological specificity factor MinE was first downregulated after 1 h of Cu treatment and upregulated from day 2 onward. The cell shape determining protein MreB was slightly upregulated between 3 and 5 days. Upregulation from 1 day onward was observed for the Z-ring protein FtsZ, with especially high Log_2_-FC on days 3 and 4 (Log_2_-FC of 3.98 and 3.62). The corresponding DNA segregation, ATPase FtsK, was significantly upregulated between 2 and 5 days of incubation. Another DNA segregation ATPase, Smc, was slightly upregulated at several time points of the resuscitation phase. The glucose-inhibited cell-division protein GidA was downregulated until 24 h of incubation but was upregulated during the resuscitation phase (days 2–5). Interestingly, a strong upregulation was observed from 2 days onward for the ParA and ParB partitioning proteins encoded by the megaplasmid pMOL30 as well as for the replication protein RepA at day 5 (Fig. S6). Such an upregulation was not observed for megaplasmid pMOL28 (Fig. S6).

### Metal stress response is active during VBNC induction and resuscitation

**Figure 5.**
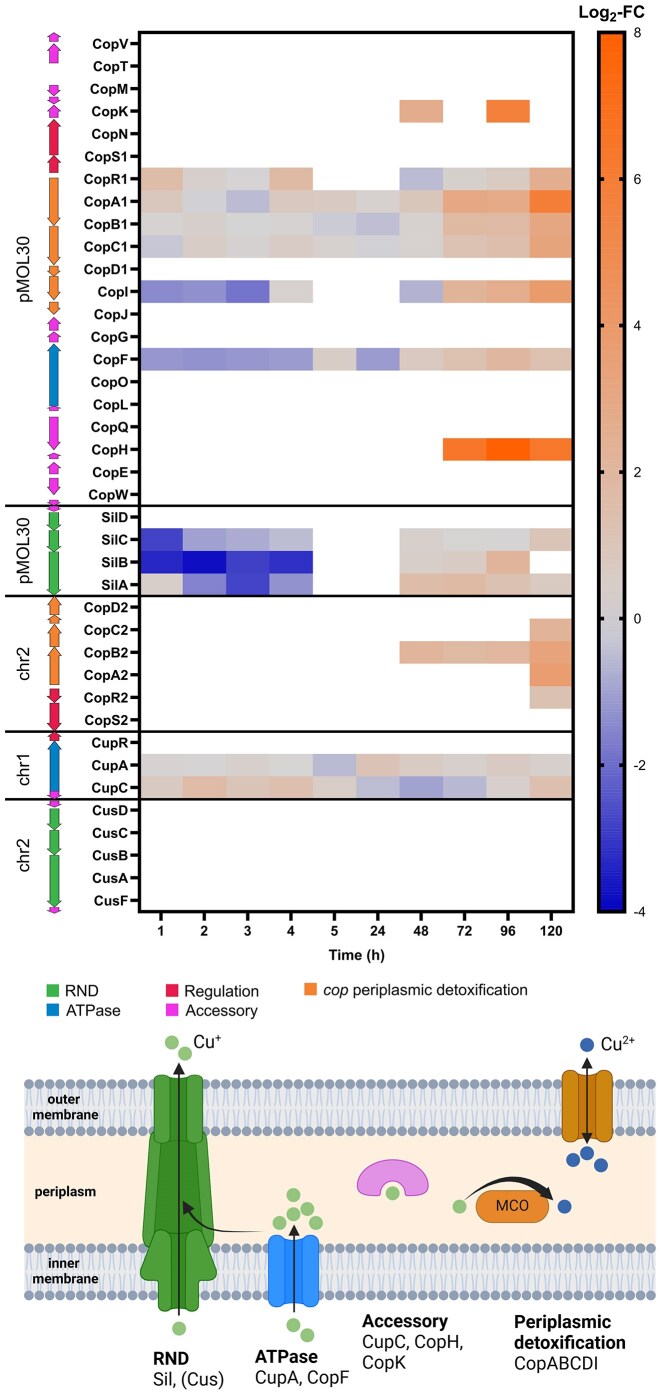
Top: log_2_-fold change (Log_2_-FC) values of cu resistance proteins (Cu-treated vs control condition) at each time point. white = not identified. arrows indicate genomic context, colors indicate (putative) function. chr1 = chromosome; chr2 = chromid. Bottom: schematic representation of cu detoxification mechanisms in CH34 (simplified). Cytoplasmic Cu^+^ ions are either directly exported via RND (resistance-nodulation-cell division family transporter) efflux systems or transported to the periplasm through ATPases. Periplasmic Cu^+^ can be bound by accessory proteins (e.g. Cu chaperones) and converted to Cu^2+^ by multicopper oxidases (MCO). CopB, an outer membrane protein, is putatively involved in the subsequent export (as well as import) of Cu^2+^. Created with BioRender.com.

*Cupriavidus metallidurans* CH34 features a multitude of metal resistance determinants that are induced by Cu stress [25, 26]. However, it is yet to be investigated whether the transition into a nonculturable state and its regulation can be linked to the activation of such cellular metal detoxification systems [6]. Differential expression was observed for several Cu detoxification systems at different time points of incubation. Their expression profiles, genomic locations and (putative) functions are summarized in Fig. 5. Firstly, the periplasmic copper detoxification system CopA_1_B_1_C_1_D_1_ (Cop1), encoded by pMOL30, as well as the chromosomally encoded homolog CopA_2_B_2_C_2_D_2_ (Cop2) were upregulated mostly at later stages of incubation (2–5 days) and non-DE or even downregulated at the beginning of Cu treatment (1–24 h). In fact, the multicopper oxidase CopA1 was one of the most induced proteins during resuscitation (Log_2_-FC of 5.90 at 5 days). The P_IB1_-type ATPase CopF and the putative cupredoxin-like copper-binding protein CopI, both encoded by pMOL30, were also upregulated at later stages (2–5 days) and strongly downregulated at early stages of Cu treatment (1–24 h). CopH and CopK, both periplasmic Cu-binding proteins also encoded by pMOL30, were strongly induced during resuscitation (e.g. CopH: Log_2_-FC of 7.92 at 4 days) but were not identified before 2 days. In addition, the three component cation efflux system SilABC, encoded by pMOL30 in proximity to Cop1, showed remarkable downregulation during the first few hours of incubation (nonsignificant for SilA) and moderate upregulation in the resuscitation phase (SilC non-DE). A dissimilar expression profile was found for the Cu chaperone CupC (upregulated until 4 h, afterward mostly non-DE), while the corresponding Cu-transporting ATPase CupA showed no significant differential expression over all time points except slight upregulation at 4 days.

**Figure 6.**
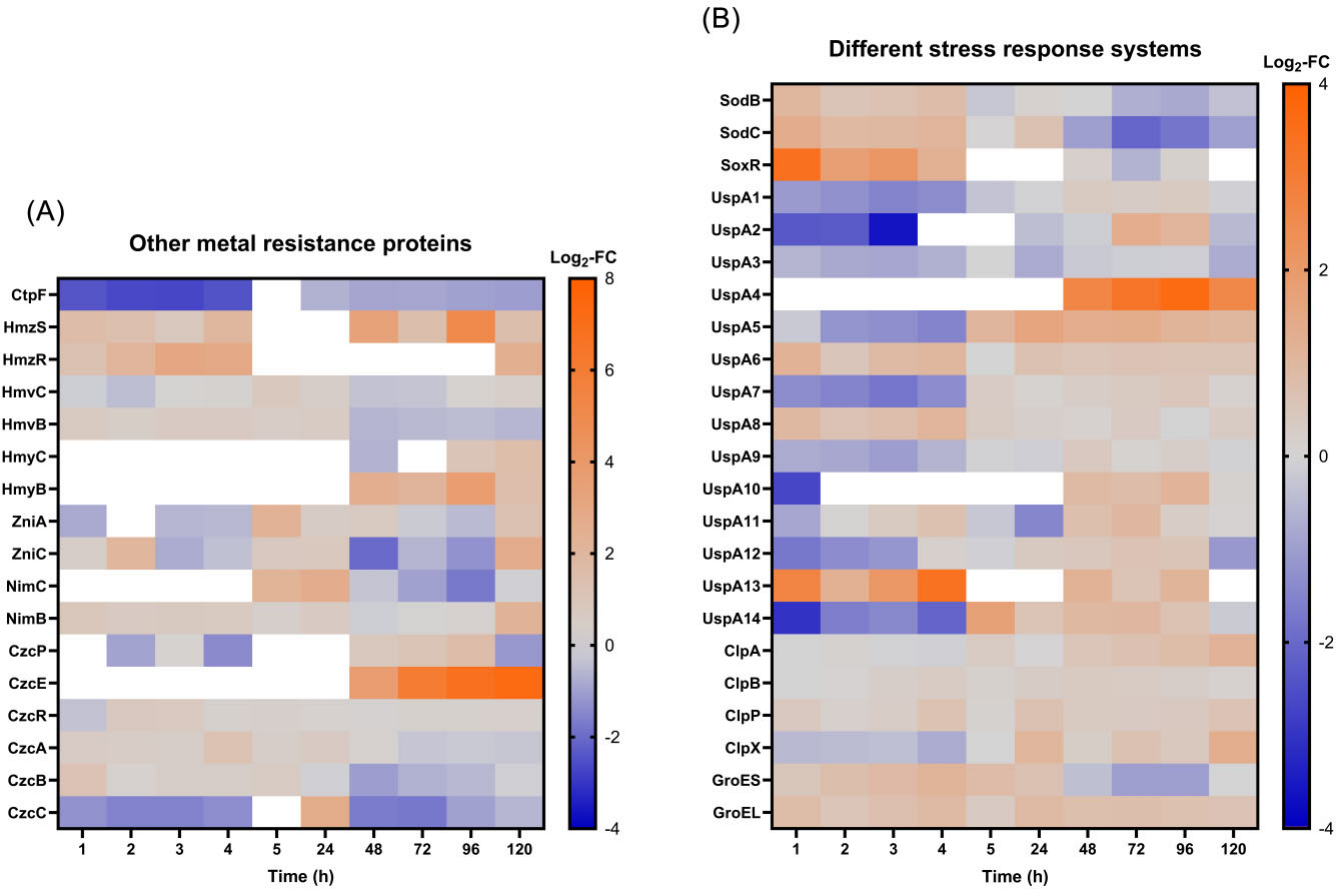
Log_2_-fold change (Log_2_-FC) values of different proteins (Cu-treated vs control condition) at each time point. White = not identified. Left: proteins linked to metal stress resistance. Right: proteins linked to different stress response systems.

We also observed sporadic induction of other metal resistance determinants in Cu-treated cells (Fig. 6A). Most notably, CzcE was strongly upregulated from day 2 onward, with Log_2_-FC ranging from 3.74 (2 days) to 7.29 (5 days) but was not identified in samples before day 2. Interestingly, other proteins of the Czc system, which are involved in Cd(II), Zn(II), Co(II) resistance, were non-DE at most time points or even downregulated (e.g. CzcC at 1, 2, 4 h and 3 days). ZniA and ZniC, parts of a tricomponent metal cation efflux system, were upregulated at 5 days while showing no significant differential expression at other time points. Moreover, HmzRS, a two-component metal response regulatory system, was upregulated at many time points, with Log_2_-FC values between 0.83 (3 h) and 5.12 (4 days). The heavy metal cation tricomponent efflux protein HmvB was slightly upregulated at 1 and 4 h of incubation (Log_2_-FC of 0.72; 0.64). Its analogue HmyB was strongly upregulated from 2 days onward but was not identified before that time point. Another heavy metal cation tricomponent efflux protein, NimB, was slightly upregulated during the first 4 h of incubation and non-DE after that (but again upregulated at 5 days). Interestingly, the cation-transporting ATPase CtpF was downregulated at almost all time points.

### Other stress response systems affected by Cu treatment

Differential expression of several systems related to general and oxidative stress response was observed (Fig. 6B). Firstly, the superoxide dismutases SodB and SodC together with the transcriptional activator for superoxide response SoxR were upregulated at several time points between 1 and 24 h of Cu exposure. Interestingly, during resuscitation (2–5 days), these proteins were non-DE or even downregulated. Conversely, many proteins of the universal stress protein UspA family (e.g. UspA5, UspA10, UspA14) were downregulated during VBNC induction (1–5 h) and were later upregulated during resuscitation. Different subunits of the ATP-dependent Clp protease were upregulated at several time points (1, 3, 5 days), and the 10- and 60 kDa chaperonines GroES and GroEL were upregulated until 4 h (GroEL even until day 5). These results could hint at the potential activation of cellular systems counteracting oxidative stress (e.g. protein modulation) imposed by Cu treatment.

### Terpenoid and cell wall biogenesis are involved in VBNC entry and exit

As Cu ions exhibit detrimental effects on bacterial cell walls, it was not surprising that several proteins linked to the COG class “Cell wall/membrane/envelope biogenesis” were DE. The outer membrane proteins Rmet_0712, Rmet_4834 and Rmet_3234 as well as the membrane-bound transglycosylases MltA and MltB were upregulated at several time points during the early incubation phase. OmpP2, a porin involved in diffusion of small hydrophilic molecules, showed strong upregulation at most time points, especially during the resuscitation phase (Log_2_-FC of 5.75 at 5 days). Notably, OmpP2 is encoded between the *cop* and *sil* clusters on pMOL30, which could indicate a possible link with Cu detoxification. The outer membrane lipoprotein Pal and the glycosyltransferase Rmet_0756 were upregulated at 1 h of Cu treatment but were downregulated between 1 and 5 days. Interestingly, the lipoprotein carrier protein LolA followed a similar trend (upregulation until 4 h, downregulation from day 2 onward). The opposite expression profile (downregulation in the VBNC induction phase and upregulation during resuscitation) could be observed for the bifunctional protein GlmU and the phospholipid synthase Cfa. Following these results, we can consider that cell wall modulation might be involved in the regulation of the Cu-induced VBNC state.

**Figure 7.**
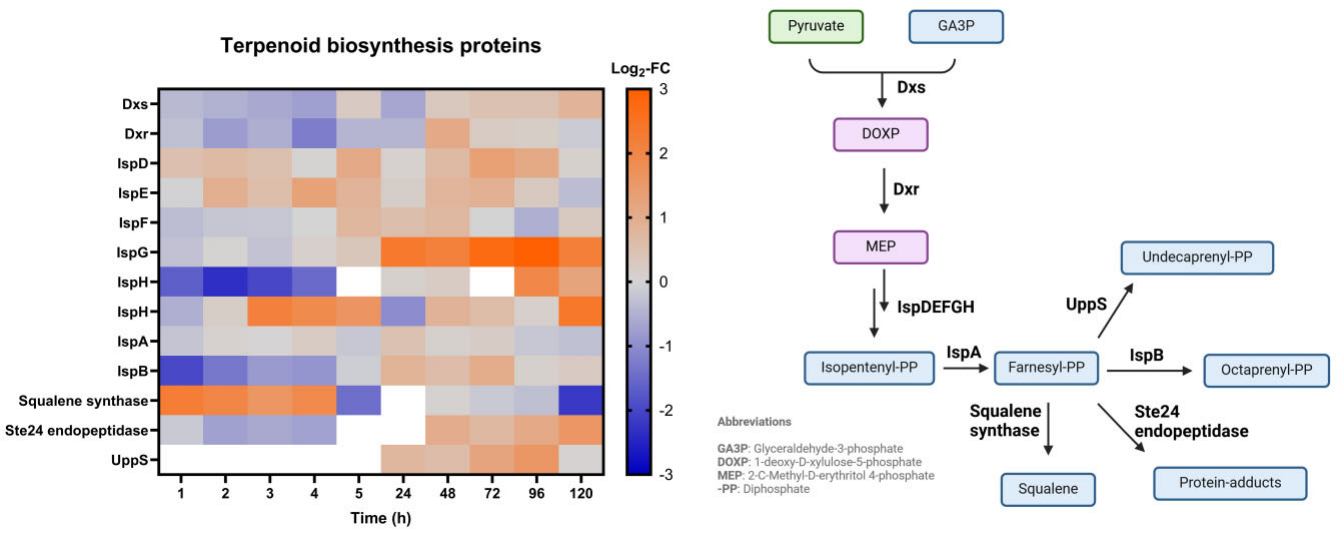
Left: log_2_-fold change (Log_2_-FC) values of proteins involved in terpenoid backbone synthesis (Cu-treated vs control condition) at each time point. White = not identified. Right: MEP/DOXP pathway (simplified). Blue = intermediate; purple = name-giving intermediate; green = important intermediate. Created with BioRender.com.

In addition, most enzymes involved in the 2-C-Methylerythritol 4-phosphate/1-deoxy-D-xylulose 5-phosphate (MEP/DOXP) pathway for the biosynthesis of isopentenyl diphosphate (IPP) were DE at different incubation phases (Fig. 7). Dxs and Dxr, catalyzing the initial reaction steps, were slightly downregulated in the first hours of incubation. Conversely, IspD, IspE, and IspF were slightly upregulated at various time points of Cu treatment. IspG was highly upregulated starting from 24 h (non-DE before that). We identified two analogues of IspH (Rmet_4169 and Rmet_2868), an enzyme catalyzing the final step of IPP biosynthesis. Interestingly, the two analogues showed diverging expression profiles during the VBNC induction phase, either strong downregulation or moderate upregulation (1–5 h). However, both were upregulated at later time points (4–5 days). IPP can be processed to different types of terpenoids via conversion to farnesyl diphosphate catalyzed by IspA (non-DE). We noticed that two enzymes of opposing downstream metabolic routes, namely the octaprenyl diphosphate synthase IspB and the squalene synthase (Rmet_5617), were showing contrasting expression profiles depending on incubation time. During the VBNC induction phase (1–4 h), IspB was downregulated while the squalene synthase was highly upregulated. During the resuscitation phase, both enzymes were mostly non-DE, however IspB was slightly upregulated at day 3, while the squalene synthase was downregulated at day 5. Moreover, other enzymes using farnesyl as substrate (UppS and Ste24 endopeptidase) were also upregulated between days 1 and 5. We can thus assume that during VBNC induction, terpenoid biosynthesis is activated and steered toward squalene formation, whereas this effect might be less relevant during resuscitation.

### Induction of the VBNC state instigates reorganization of carbon metabolism

#### Polyhydroxybutyrate metabolism

**Figure 8.**
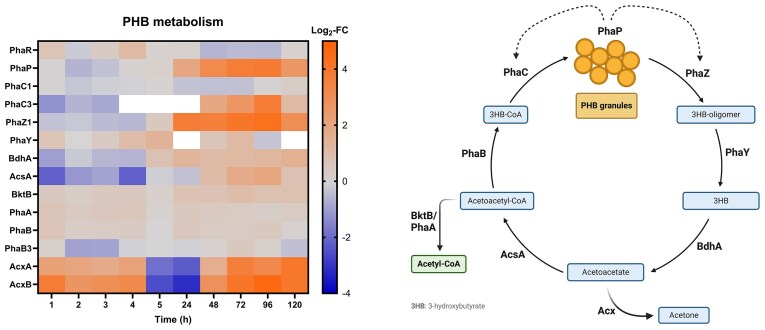
Left: Log_2_-fold change (Log_2_-FC) values of proteins involved in polyhydroxybutyrate (PHB) metabolism (Cu-treated vs control condition) at each time point. White = not identified. Right: PHB cycle (simplified). Blue = intermediate; green = potential product, feeding into subsequent pathway. Created with BioRender.com.

Since stationary-phase cells were suspended in mineral water without additional nutrients, the question arose what kind of energy resources are being used to establish (partially) strong differential protein expression over the course of several days. Subsequently, we further investigated whether transition into the VBNC state also correlated with changes in the central carbon and energy metabolism. Differential expression of proteins linked to PHB metabolism, used as an energy reserve during carbon-limiting conditions [27], was found during the resuscitation phase (Fig. 8). The PHA-granule associated protein Phasin was strongly upregulated from 1 day onward. Phasins participate in PHB synthesis and degradation through activation of both PHB polymerases and depolymerases [28]. Accordingly, we monitored upregulation of both, polymerase PhaC3 and depolymerase PhaZ1 between 1 and 5 days (PhaC1 was non-DE). Both proteins were non-DE during the VBNC induction phase. It was shown for *Cupriavidus necator* that phosphorylation of PHB poly- and depolymerases is involved in their regulation [29]. It is likely that a similar effect is present here to prevent simultaneous build-up and break-down of polymers. The 3-hydroxybutyrate oligomer hydrolase PhaY and the D-beta-hydroxybutyrate dehydrogenase BdhA, involved in subsequent catabolic reaction steps resulting in the formation of acetoacetate, were slightly upregulated (BdhA only during resuscitation), hinting at a net-flux toward PHB degradation. From here, two different metabolic routes are possible.

Firstly, acetoacetate can be further processed to two equivalents of Acetyl-CoA (AcCoA) through the AcCoA synthetase (AcsA) and the AcCoA acetyltransferase (BktB), both of which were upregulated between 1 and 5 days (AcsA was downregulated in the first hours of incubation). The intermediate product acetoacetyl-CoA could also be fed back toward PHB synthesis through the enzyme PhaB, which however was not differentially expressed (DE). Net-flux will therefore end up in the formation of AcCoA, to deliver a metabolically pluripotent compound, as well as to avoid a futile cycle of PHB synthesis and degradation.

Secondly, acetoacetate can be converted to acetone via the acetoacetate decarboxylase, however this enzyme was not found in our data, nor is it mapped for *C. metallidurans* in the KEGG database. Decarboxylation could also theoretically happen through the reverse reaction catalyzed by the acetone carboxylase with concomitant ATP formation. Notably, the acetone carboxylase subunits AcxAB were found highly upregulated throughout most sample time points (downregulated only at 5 and 24 h), hinting at a potential involvement of the enzyme in this pathway. Subunit AcxC and the corresponding regulator AcxR were only identified at 5 and 24 h. Deletion of *acxR* (which resulted in the loss of acetone carboxylase production and growth on acetone or isopropanol [30]; Fig. S7) resulted in a higher proportion of cells entering the VBNC state compared to the parental strain but did not affect the overall resuscitation behavior. It is therefore possible that besides AcCoA production, acetoacetate is used for the formation of ATP during increased energy requirements of cells facing Cu stress. However, literature is not clear about whether this reaction is favorable in physiological conditions, and acetoacetate decarboxylation was even reported to happen spontaneously in aqueous solution [31].

**Figure 9.**
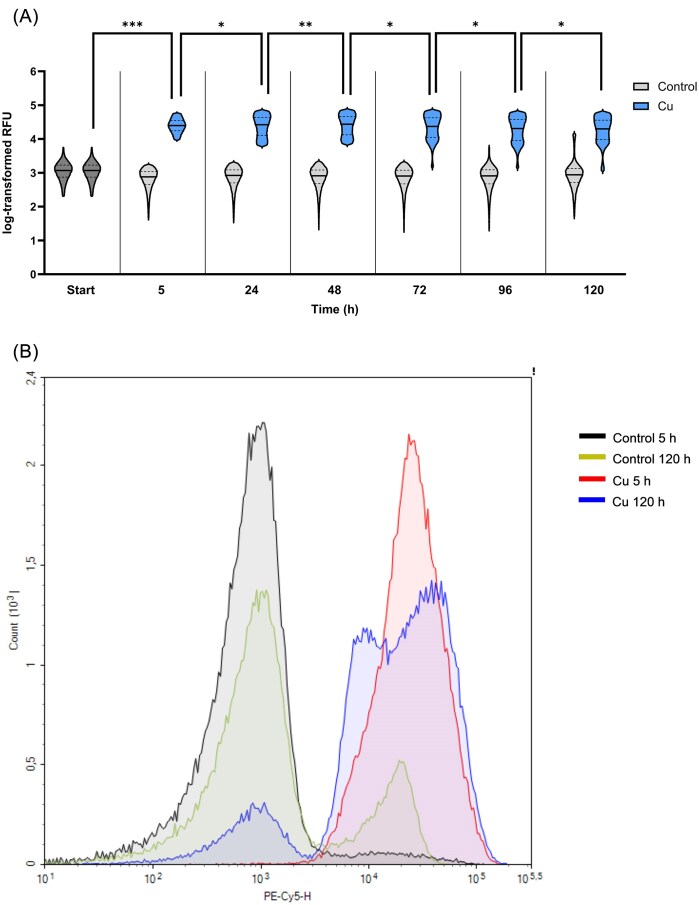
(A) Cu-treatment caused fluorescence shifts (Nile red staining) compared to control cells, but fluorescence intensity changed only slightly with time. Log-transformed relative fluorescence units (RFU) of events falling into the 10–90-interpercentile range were plotted. Median RFU values are represented as straight lines, quartiles as dotted lines. Effect sizes calculated by cramér's V: *small, **medium, ***large. (B) Histogram overlay for samples at 5 and 120 h.

Nonetheless, following these results, we can contemplate that through degradation of PHB granules, a pool of AcCoA is generated that might be used to reactivate the central carbon metabolism of dormant cells. This consideration prompted us to investigate whether intracellular PHB levels are different before and after resuscitation. To this end, the PHB content of cells was quantified via a Nile red assay [32, 33]. The median RFU (mRFU), which correlates with intracellular PHB levels and can thus be used for relative quantification [34], was 1170 for the starting condition. In addition, control cells had relatively constant fluorescence over all time points (mRFU between 758 and 878). Using Mood's median test, we could verify that mRFU values were significantly higher for Cu-treated cells at all time points (Table 2). However, due to the very large number of recorded events, we resorted to the estimation of effect sizes in the Cu-treated condition using Cramér's V [18]. A high effect size was detected by comparing the 5-h Cu sample to the starting condition (Fig. 9A), suggesting a relative change in PHB levels. This result was surprising as our proteomics data showed no considerable upregulation of PHB metabolizing enzymes in the first few hours of Cu treatment. Mostly, low effect sizes were detected when comparing Cu-treated samples at adjacent time points (Table 2, Cu_T_ vs Cu_T+1_), indicating only small correlations between incubation time and changes in PHB levels. One single medium effect size between 24 and 48 h of Cu treatment could however corroborate the increased expression of PHB metabolizing enzymes starting at these time points (Fig. 8). While the log-transformed RFU of cells at 5 h of Cu treatment seemed to follow a normal density function, transition to a bimodal distribution could be observed starting from 24 h, with skew toward lower RFU values (Fig. 9A and B). Cu-treated cells clearly show a more heterogenous composition at 120 h than at 5 h, suggesting that a subpopulation with lower PHB content is present (Fig. 9B). This could indicate the emergence of a subpopulation that more actively metabolizes PHB, corroborating the strong upregulation of PHB degrading enzymes observed during resuscitation. Interestingly, a subpopulation with higher RFU was detected at 120 hours in the control condition, with an inverted proportion of cellular events compared to the Cu sample (Fig. 9B). Therefore, PHB synthesis in nontreated cells could be active to a similar extent as degradation in treated cells after several days of incubation in mineral water.

**Table 2.** Median relative fluorescence units (mRFU) and standard deviations of samples stained with Nile Red at different time points

	Time (h)						
	Start	5	24	48	72	96	120
mRFU Control	1170	758	838	821	808	814	878
Std. deviation	796.8	411.5	475.1	494.0	481.7	683.5	2208
mRFU Cu	1170	25 013	26 394	27 393	23 532	20 462	19 997
Std. deviation	796.8	11 956	18 043	19 094	18 898	17 406	16 563
*P-*value	–	<.001	<.001	<.001	<.001	<.001	<.001
Cramer's V	0.93	0.03	0.30	0.06	0.05	0.01	

*P*-values from Mood's median test (Cu vs Control) and Cramér's V (Cu Time vs Cu Time + 1) values are given.

#### Glyoxylate cycle and gluconeogenesis

**Figure 10.**
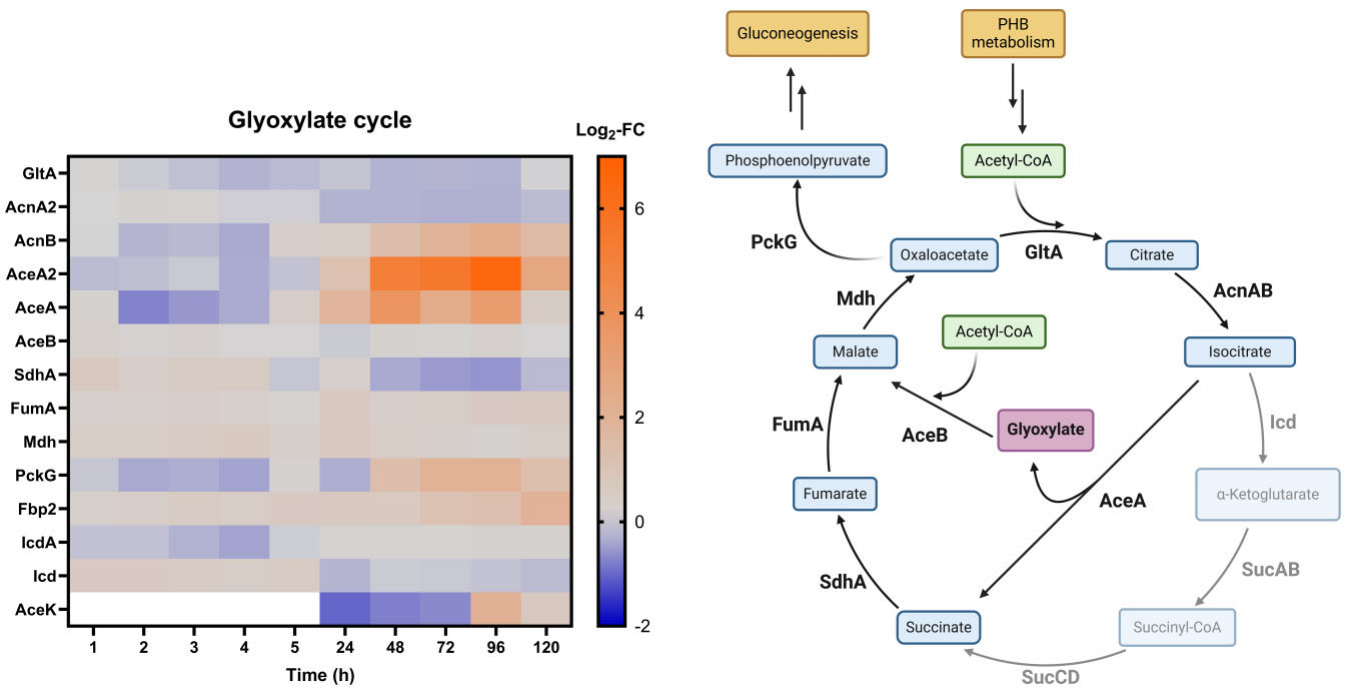
Left: Log_2_-fold change (Log_2_-FC) values of proteins involved in the glyoxylate cycle (Cu-treated vs control condition) at each time point. White = not identified. Right: glyoxylate cycle (simplified). Blue = intermediate; purple = name-giving intermediate; green = feeding into the cycle; yellow = related pathway. Created with BioRender.com.

Cells in the resuscitation phase showed high upregulation of both isocitrate lyase homologues AceA and AceA2 (Fig. 10, particularly high AceA2 expression e.g. 6.53 Log_2_-FC at 4 days). Upregulation of this enzyme is an indicator for the glyoxylate cycle, which bypasses the two decarboxylation steps of the citric acid cycle to activate carbohydrate synthesis [35, 36]. Precursors of the glyoxylate cycle are oxaloacetate and AcCoA, the latter can be supplied from PHB degradation. Condensation of these two molecules catalyzed by the citrate synthase (GltA, non-DE) yields citrate (identical reaction to the citric acid cycle). Citrate can be converted to isocitrate by the aconitase Acn. AcnB was upregulated from day 2 onward (AcnA was non-DE). The highly upregulated isocitrate lyase cleaves isocitrate into succinate and glyoxylate. The second enzyme that is characteristic of the glyoxylate cycle, the malate synthase (AceB), uses glyoxylate and another equivalent of AcCoA to form malate. Even though AceB was non-DE, the bypass is driven forward since this reaction step is thought to be irreversible [37]. The malate dehydrogenase Mdh (slightly upregulated at 4 and 24 h, else non-DE) closes the cycle by converting malate to oxaloacetate. The formed succinate is simultaneously converted to another equivalent of oxalacetate [35]. Subsequently, it can be further processed to phosphoenolpyruvate through the phosphoenolpyruvate carboxykinase PckG, which was upregulated between days 2 and 5 (non-DE at time points before day 2). This marks the first step of gluconeogenesis. Catalyzing the rate-determining step of gluconeogenesis, the fructose-1,6-bisphosphatase Fbp2 was also upregulated from day 2 to 5. In addition, many other gluconeogenetic enzymes (e.g. GpmB, Pgk, CbbG, CbbA; Fig. S8) followed the same expression profile (upregulation during resuscitation, non-DE during VBNC induction).

Meanwhile, the competing isocitrate dehydrogenase (Icd), feeding into the citric acid cycle, was mostly non-DE. Interestingly, the corresponding kinase AceK was upregulated at days 4 and 5 (not detected or no significant differential expression before that). These results suggest that the glyoxylate shunt and subsequent gluconeogenesis are active during resuscitation.

#### Pyruvate metabolism

**Figure 11.**
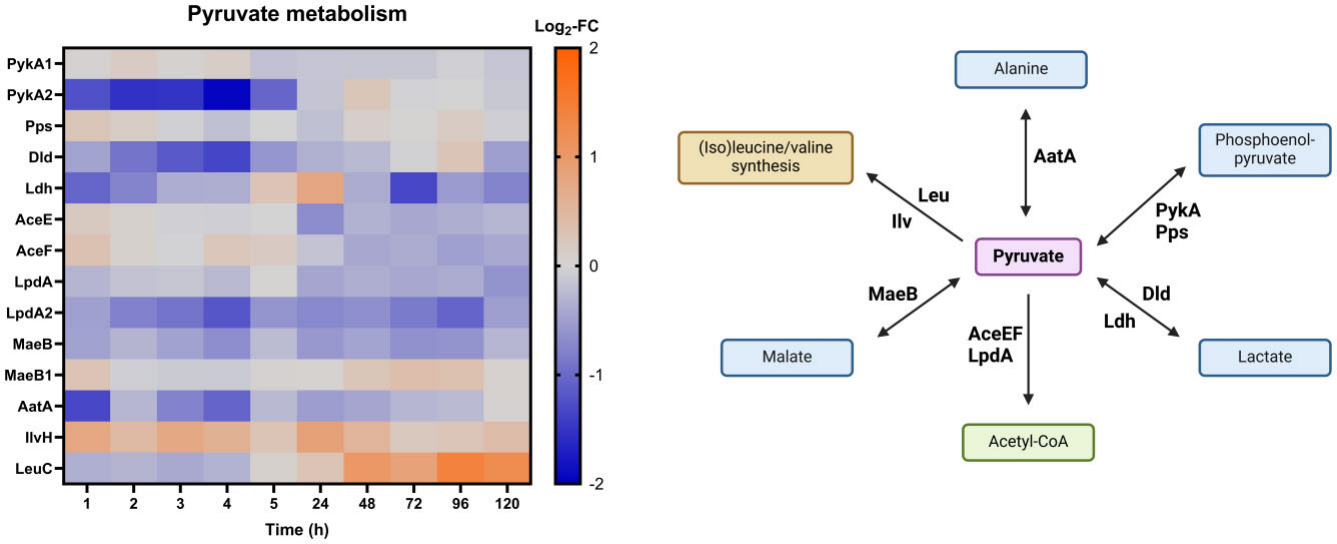
Left: log_2_-fold change (Log_2_-FC) values of proteins involved in pyruvate metabolism (Cu-treated vs control condition) at each time point. Right: possible conversions of pyruvate (simplified). Blue = conversion product; purple = name-giving intermediate; green = important intermediate; yellow = related pathway. Created with BioRender.com.

Many pyruvate-metabolizing enzymes were significantly downregulated in VBNC and resuscitating cells (Fig. 11). The pyruvate kinase PykA2, converting phosphoenolpyruvate to pyruvate, was distinctly downregulated until 5 h of Cu treatment (however, PykA1 and Pps were non-DE). Notably, we also observed decreased expression of enzymes involved in the interconversion of pyruvate and AcCoA: AceE (downregulated from 24 h onward) and LpdA2 (downregulated at most time points). Downregulation of these enzymes could be an important mechanism to avoid interference with the glyoxylate cycle. In addition, Ldh and Dld, enzymes involved in pyruvate-lactate conversions, as well as the malate dehydrogenase MaeB were downregulated at several time points (MaeB1 was non-DE). The aspartate aminotransferase AatA, catalyzing alanine-pyruvate conversion, was downregulated during the first few hours of incubation. Conversely, several enzymes of the Ilv and Leu families involved in the synthesis of branched amino acids were upregulated throughout Cu treatment, suggesting that adaptations in amino acid availability might be required at different stages of the Cu-induced VBNC state.

#### Vitamin biosynthesis—thiamine supplementation prevents VBNC formation

**Figure 12.**
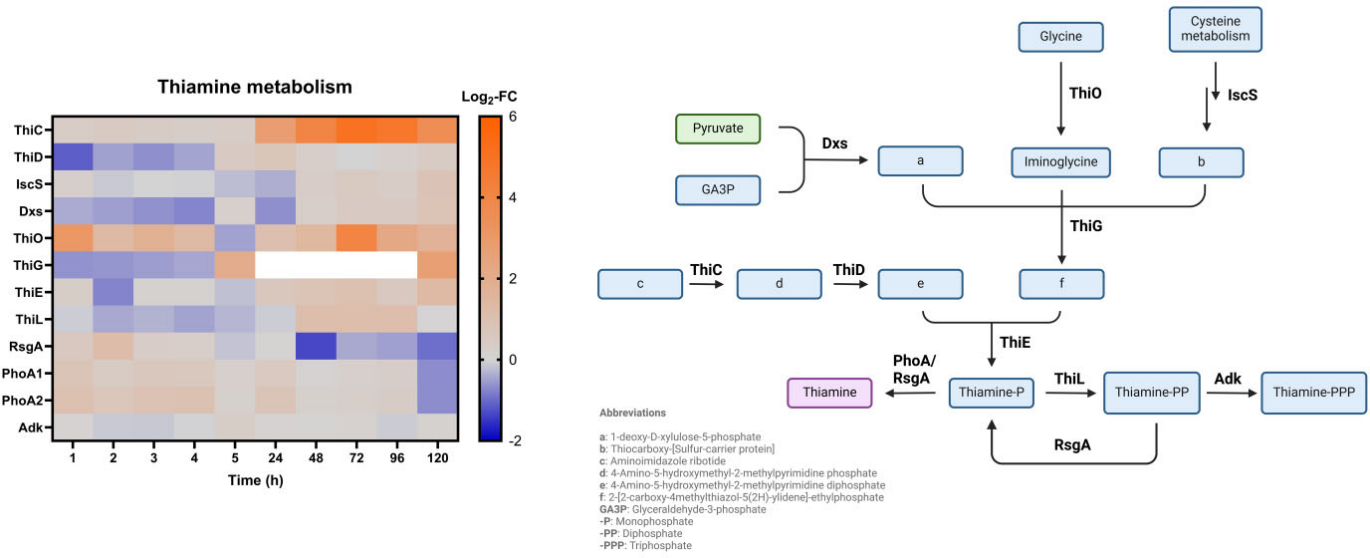
Left: Log_2_-fold change (Log_2_-FC) values of proteins involved in thiamine metabolism (Cu-treated vs control condition) at each time point. White = not identified. Right: thiamine biosynthesis pathway (simplified). Blue = intermediate; purple = name-giving intermediate; green = important intermediate. Created with BioRender.com.

In our proteomic analysis, proteins involved in thiamine biosynthesis were found upregulated especially during the resuscitation phase (Fig. 12). Most notably, ThiC and ThiO were upregulated between 1 and 5 days of Cu exposure (ThiO also between 1 and 4 h), and ThiE was slightly upregulated at these time points. The thiazole synthase ThiG was upregulated at 5 days but was not identified or non-DE in samples before that. ThiD was downregulated in the VBNC induction phase (between 1 and 4 h) and slightly upregulated afterward at 1 day of incubation (non-DE during the later phase). Other enzymes in the thiamine biosynthetic pathway (Dxs, IscS) were slightly upregulated only at 5 days (Dxs was also downregulated during the VBNC induction phase). The pathway leads to the formation of thiamine monophosphate, which can be further processed to thiamine pyrophosphate by the thiamine monophosphate kinase ThiL. ThiL was upregulated between 2 and 4 days, while the competing phosphatases PhoA and RsgA were non-DE or slightly downregulated. Interestingly, PhoA was upregulated at several time points until 1 day. In addition, many enzymes of the biotin synthesis pathway (BioABCDF, FabGI) were upregulated between 2 and 5 days but non-DE or downregulated at earlier time points, hinting at a potential role of this vitamin in resuscitation.

Thiamine had previously been tested as a compound with potential resuscitation-promoting properties [38]. Interestingly, supplementing mineral water with 5.6 mM thiamine at the start of incubation impeded loss of culturability in CH34 cells upon Cu treatment (Fig. S9A). This result suggests that abundant thiamine might prevent CH34 from entering the VBNC state. It is possible that this effect is caused by thiamine-mediated sequestration of Cu ions. However, when fully grown CH34 was challenged with a lethal dose of 5 mM CuSO_4_ for 2 h, thiamine addition (equimolar) did not lower the toxic effect (Fig. S9B).

## Discussion

Water-borne cells of *C. metallidurans* CH34 were previously reported to enter the VBNC state upon Cu exposure [13]. To further characterize this phenomenon, we extended the sample time points and confirmed reproducibility in a different water source. Shortly after the start of the Cu treatment, approximately 99.99% of cells lose their culturability. Coincidently, only approximately 10% of the cells lose membrane integrity during the first 5 h of incubation, indicating that the majority of nonculturable cells are still intact, i.e. VBNC. Therefore, VBNC cells cannot be distinguished from culturable cells by such a membrane permeability assay. The increase of the nonviable fraction at 24 h most likely stems from cells that are unable to resuscitate from the VBNC state.

A shift from VBNC to the resuscitation phase could potentially be determined already after 5 h of Cu exposure. This time point shows a distinctly low number of up- and down-regulated proteins, which might reflect a reset of the cellular proteome mediating resuscitation. Accordingly, proteomic adaptations were most discernible either between 1 and 4 h or after 24 h of Cu treatment. For example, the COG classes “Cell cycle control, cell division, chromosome partitioning,” “Translation, ribosomal structure, and biogenesis,” and “Cell motility” contained particularly high proportions of upregulated proteins (around 50% of categorized proteins were upregulated), especially during resuscitation. In general, downregulated proteins were less abundant in these classes.

Strong oxidative stress, e.g. mediated by redox-active transition metals like Cu, can cause heavy damage to ribosomes [39]. We noticed that many RPs, represented in the COG class “Translation, ribosomal structure, and biogenesis,” were upregulated at most time points. Upregulation of RPs during the early incubation phase (1–4 h) could possibly counteract oxidative damage to secure unimpaired translation, which in turn is required for a proper adaptive response to the stressor. Interestingly, during the resuscitation phase (2–5 days), at least 80% of identified RPs were upregulated. A similar observation has been reported for *E. coli*, where the cellular ribosome content was linked to the resuscitation rate [40]. Notably, enrichment of the ribosome KEGG-pathway has been monitored for *Acidovorax citrulli* cells resuscitating from a Cu-induced VBNC state [41]. It can thus be argued that upregulation of RPs is needed to counteract acute oxidative stress and to help with enhanced translational requirements during resuscitation. One of such requirements could, for example, be the expression of proteins from the “Cell cycle control, cell division, chromosome partitioning” COG class. In fact, several proteins linked to cell division were upregulated during resuscitation. This could imply that resuscitating cells are establishing a set of proteins that allow them to initiate regrowth once favorable conditions are reached. In this regard, the strong upregulation of pMOL30-encoded proteins involved in the plasmid's partitioning and replication could hint at the need for appropriate maintenance and stability of pMOL30 as it carries metal determinants that play an important role during resuscitation.

Cu resistance determinants encoded on pMOL30 include the periplasmic detoxification system Cop1, the inner membrane P_IB1_-type ATPase CopF and the RND (resistance-nodulation-cell division) metal efflux system SilABCD, for all of which respective chromosomally encoded homologues exist (Cop2, CupA, Cus) [26, 27]. Differential expression of these systems (except Cus—not detected) was monitored throughout the Cu-induced VBNC state. Firstly, Cop1 proteins were generally upregulated in resuscitating cells (2–5 days, CopD1 was not detected) but not during the early stage of Cu treatment (1–24 h, mostly non-DE). This result stands in stark contrast to previous reports describing acute induction of the *cop* system in CH34 facing Cu stress [25, 42–44]. However, in these studies, Cu treatment was performed in growth medium, while in our experiment, water-borne cells of CH34 were exposed to Cu^2+^ ions without addition of nutrients. Lack of energy resources could therefore be the reason for the delayed expression of proteins that are potentially vital for culturability. High upregulation of Cop1 at the later time points suggests that periplasmic detoxification is an important mechanism during resuscitation. This result is corroborated by the upregulation of the chromosomally encoded Cop2 proteins at 5 days (mostly not detected at other time points). Periplasmic detoxification could be aided by the putative cupredoxin-like copper-binding protein CopI, as well as CopF, an ATPase capable of transporting Cu ions to the periplasm. Both of these proteins were upregulated during the resuscitation phase but downregulated at early sample time points, hinting at impaired Cu ion export during the VBNC phase. Strong downregulation was also monitored for the chromosomally encoded cation-transporting ATPase CtpF, while CupA was mostly non-DE. Moreover, early phase downregulation of the Sil system with slight upregulation starting at 2 days of incubation supports the assumption that Cu ion export might be stalled in VBNC cells and activated during resuscitation.

In addition, the plasmidic Cop system also contains several chaperone-like accessory proteins capable of binding Cu ions, however only two of them were identified in our data. CopK and CopH both showed drastic upregulation during resuscitation but were not found at earlier time points. CopK is a small periplasmic protein capable of binding Cu^+^ and Cu^2+^ at separate sites and is thought to act as a scavenger for periplasmic Cu-ions, delivering them to outer membrane proteins for efflux [45]. CopH is a periplasmic, dimer-forming protein capable of binding 2 Cu ions per dimer and was reported to show significant homology to CzcE [46]. Notably, the pMOL30-encoded protein CzcE showed the third highest Log_2_-FC (7.29) among all identified proteins (after CopH and PilJ). This protein had first been characterized to be induced by zinc and linked to a regulatory function in cobalt, zinc, and cadmium resistance in CH34 [47]. It was later determined to be a periplasmic Cu-binding protein, putatively involved in Zn-Cu cross regulation [48]. These proteins could potentially have a supportive function in periplasmic detoxification by sequestering accumulating Cu ions, thus preventing further cellular damage, and conveying them toward export systems. Moreover, a potential cross-regulatory function of CzcE with the Cop system is conceivable since no remarkable upregulation of other Czc proteins was monitored. Intriguingly, the Cop regulator CopR1 was only sporadically upregulated (1 and 4 h, 5 days), supporting the hypothesis that other regulatory systems are involved in the induction of Cop proteins. The HmzRS regulatory system, which was upregulated throughout Cu exposure, was reportedly linked to metal resistance due to its similarities to the Czc metal efflux system [49]. A putative regulatory involvement of CzcE and HmzRS can thus not be ruled out.

The present data suggests that despite CH34’s high level of metal resistance, it is not able to remain in a culturable state when Cu stress is combined with nutrient depletion. Although we observed short-term upregulation of metal efflux pumps (e.g. HmvB, NimB), this could be a futile effort since no sequestration of Cu-ions takes place. The cells might thus opt to accumulate Cu ions and transition into the VBNC state to sustain viability. Once certain amounts of Cu-binding proteins are expressed and located in the periplasm, cells might be able to sustainably deal with environmental Cu stress. Subsequently, Cu export systems could be activated, allowing resuscitation to happen. Elevated Cu concentrations can have diverse toxic effects on different cellular components, e.g. oxidative stress or mismetalation of cuproenzymes [50]. It can therefore be expected that the Cu-induced VBNC state is paralleled by a broader cellular stress response than solely the activation of metal detoxifying systems.

Our data shows that also other stress response systems are DE during the different VBNC phases. Firstly, we observed upregulation of terpenoid biosynthetic enzymes including the squalene synthase. It could be possible that CH34 cells produce squalene to counteract elevated levels of oxidative stress during the first few hours of Cu treatment. Several studies attest the antioxidant properties of this biomolecule [51, 52]. Squalene has also recently been found accumulated in VBNC cells of *Campylobacter jejuni* [53]. Moreover, upregulation of superoxide dismutases SodB and SodC, their regulator SoxR, several universal stress proteins (UspA family) as well as different protein-modulating systems like chaperonins or the Clp protease, suggests that oxidative stress remediation might be activated, especially during early stage Cu treatment. In the proteomic response of *Micrococcus luteus* cells in a nutrient depletion-induced VBNC state, UspA and superoxide dismutase were detected, among others [54]. Interestingly, in a transcriptional study investigating the Cu-induced VBNC state in *Xanthomonas campestris*, overexpression of Clp-related genes was linked to VBNC induction [55]. Our results support the theory that distantly-related bacteria might share certain conserved proteins associated with dormancy. Such conserved proteins might not even depend on the kind of stressor. For example, the Clp protease as well as chaperonins GroES and GroEL were DE in CH34 cells in a VBNC state induced by desiccation and starvation [56]. The same study reports differential expression of the LolA and GlmU proteins, both of which were also DE in our data. Besides LolA and GlmU, several other DE proteins were linked to the ‘Cell wall/membrane/envelope biogenesis’ COG class, which seemed to be activated during the VBNC induction phase.

Another interesting observation was increased motility in Cu-treated cells. Several proteins of the Che and Pil families, as well as flagellin FliC2 were upregulated throughout Cu exposure. Higher abundance of such motility proteins could lead to increased formation of pili and/or flagella, potentially mediating the enhanced swarming potential of resuscitated cells. This is an interesting aspect as a shift toward higher motility would not be promptly expected for “dormant” cells. In natural habitats, CH34 might activate chemotaxis when facing Cu stress to facilitate migration toward less harmful environments. Enhanced cell motility could thus be important to reach favorable conditions where resuscitation can happen and might be tightly coordinated with Cu homeostasis. In a proteomic study of *Vibrio cholerae* VBNC cells, proteins linked to bacterial chemotaxis were reported to be accumulated [57]. Chemotaxis proteins were also enriched in resuscitating *Acidovorax citrulli*cells following a Cu-induced dormant state [41]. Our observations further strengthen the link between chemotaxis and the VBNC state.

During the VBNC state and resuscitation, CH34 showed significant alterations in its central carbon and energy metabolism. Firstly, cells transitioning to VBNC particularly showed downregulation of pyruvate metabolizing enzymes. This result corresponds with the frequently reported link between the VBNC state and slowed-down carbon metabolism [11, 56, 58–60]. We suggest that reactivation takes place via the accumulation of AcCoA produced via degradation of intracellular PHB granules. Most enzymes part of this metabolic pathway were found upregulated in cells after 1 day of Cu treatment until full resuscitation at day 5. PHB degradation in Cu-induced VBNC cells had already been observed for the plant pathogen *Ralstonia solanacearum* [61]. In the closely related *Cupriavidus necator*, PHB accumulation was reported to prevent entry and/or to help recover from the VBNC state induced by low temperature [62]. Obruca *et al*. showed that the monomer of PHB, 3-hydroxybutyrate (3HB), has protective effects on enzymes in conditions such as Cu-induced oxidative stress [63]. The authors also suggest that intracellular accumulation of this metabolite might increase stress resistance by acting as a chemical chaperone. Based on our proteomics data, we need to consider that further processing of 3HB to AcCoA and subsequent feed into the glyoxylate cycle is probably favored in CH34. What remains unclear is whether PHB itself is formed before or during the transition into VBNC. When stationary-phase cells were stained with Nile Red, the mRFU (correlating with intracellular PHB levels) stayed relatively constant during 5 days of incubation in mineral water. In the Cu condition, the value increased approximately 20-fold after only 5 h of incubation, suggesting a drastic accumulation of PHB during VBNC formation. However, it could be possible that existing PHB granules are simply made more accessible to the dye as a consequence of Cu ion stress (e.g. by membrane permeabilization). This artifact seems even more likely considering that no remarkable upregulation of PHB metabolizing enzymes could be detected during the first few hours of incubation. However, upregulation of such enzymes was indeed observed during the resuscitation phase, and it is likely that a PHB-degrading subpopulation is present. We can thus assume an association between PHB degradation and restoration of culturability in CH34, even though effect sizes were low for most time points.

Breakdown of intracellular PHB leads to a growing pool of AcCoA that could be used for a shift toward anabolic metabolism. This is done via activation of the glyoxylate cycle. The key enzyme for this pathway, isocitrate lyase AceA, was strongly upregulated in resuscitating cells. The glyoxylate cycle might be a mechanism to trigger resuscitation, bypassing the catabolic steps of the citrate cycle to activate gluconeogenesis. Accordingly, we monitored upregulation of the phosphoenolpyruvate carboxykinase PckG (which is linking the glyoxylate cycle to gluconeogenesis) and the rate-limiting gluconeogenic enzyme Fbp2 in resuscitating cells. The glyoxylate cycle has already been linked to the VBNC state in *M. luteus*and was hypothesized to help nutrient-deprived VBNC cells survive [54]. A functional glyoxylate cycle has recently been described as a crucial metabolic pathway for viability in *Pseudomonas aeruginosa* VBNC cells [59, 64]. In addition, we found that enzymes involved in the biosynthesis of biotin and thiamine were upregulated, cofactors that are important for various reactions of carbohydrate metabolism [65, 66]. Thiamine supplementation to mineral water largely prevented VBNC formation and its biosynthesis was activated during resuscitation. This suggests that thiamine abundance could be an important factor for cell culturability. While stimulation of carbohydrate metabolism might be an explanation for this effect, it is also possible that VBNC-inducing Cu ions are simply sequestrated by thiamine, for instance through chelation by its thiazole moiety. However, no evidence for a thiamine-mediated decrease in bioavailable Cu levels could be obtained so far.

## Conclusion

This study provides an in-depth, time-resolved look into the proteomic state of water-borne *C. metallidurans* CH34 transitioning to and resuscitating from a Cu-induced VBNC state. We found wide-ranging adaptations in the proteomes inherent to these unique cell states, including but not limited to the activation of Cu detoxification systems. While those played a crucial role during the resuscitation phase, a more general response to oxidative stress, comprising the enhanced production of RPs and chaperones, as well as cell wall modulations, were observed in the VBNC entry phase. Notably, induction of the VBNC state is correlated with downregulation of pyruvate metabolism. Our data suggests that central carbon and energy metabolism were gradually reactivated through degradation of intracellular PHB, yielding AcCoA, which can subsequently be used for gluconeogenesis via the glyoxylate shunt. Newly formed cellular resources could then possibly be used to deliver the energy needed for increased production of Cu-detoxifying proteins (e.g. Cop proteins), permitting resuscitation from the VBNC state. Furthermore, we also detected an increased expression of chemotaxis-related proteins in Cu-treated cells that was verified phenotypically through motility assays. To further investigate mechanisms that are controlling the VBNC state, we propose integrating metabolomic and proteomic data, which would improve our fundamental understanding of bacterial dormancy. This could help to identify potential weak-points in a unique bacterial survival strategy.

## Supplementary Material

mfaf007_Supplemental_Files

## Data Availability

All raw mass spectrometry proteomics data have been submitted to the ProteomeXchange Consortium [67] via the PRIDE [68] partner repository with the dataset identifiers PXD056297, PXD056296, and PXD056326.
