# Supplementary material for: Time-resolved proteomic profiling of Cupriavidus metallidurans CH34 in the copper-induced viable-but-nonculturable state
Source: Metallomics. 2025 Feb 17;17(3):mfaf007. doi: 10.1093/mtomcs/mfaf007 (PMC11886801; doi:10.1093/mtomcs/mfaf007)
Supplement: mfaf007_Supplemental_Files [file mfaf007_supplemental_files.zip › Suppl_data_Supplementary_Figures.docx]

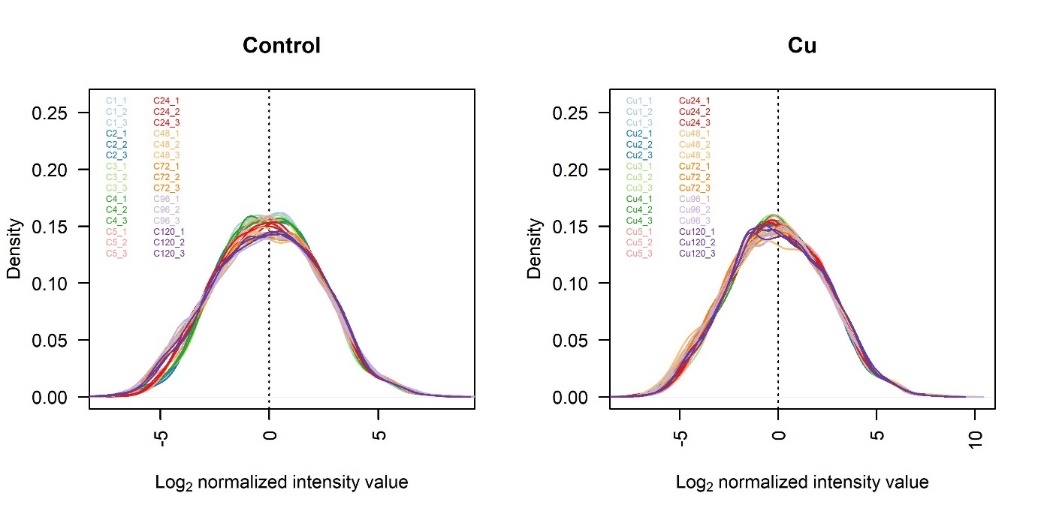


Supplementary Figure S1: Density plots of Log_2_ normalized intensity values (from protein mass spectrometry) of all replicates from all time points. Left: control condition. Right: Cu condition.


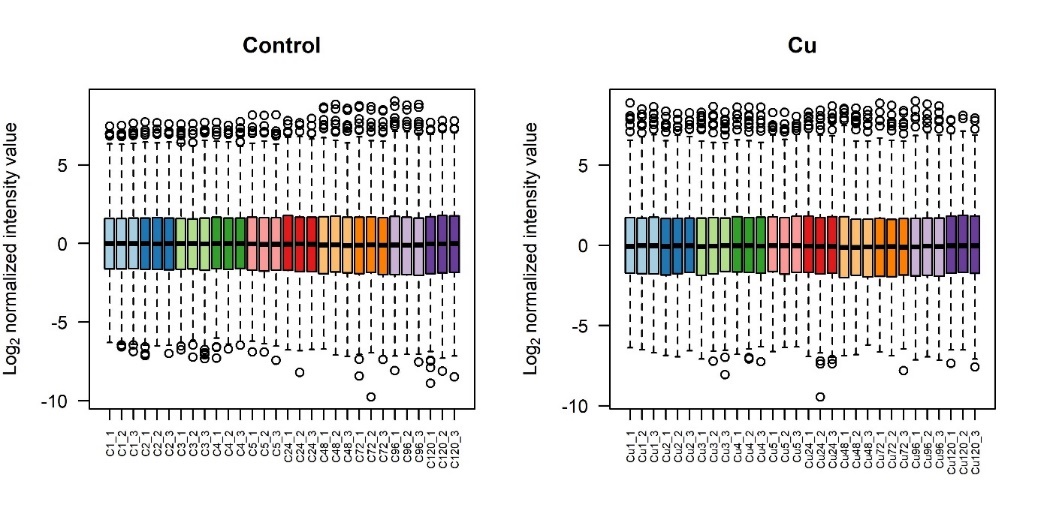


Supplementary Figure S2: Box plots of Log_2_ normalized intensity values (from protein mass spectrometry) of all replicates from all time points. Left: control condition. Right: Cu condition.


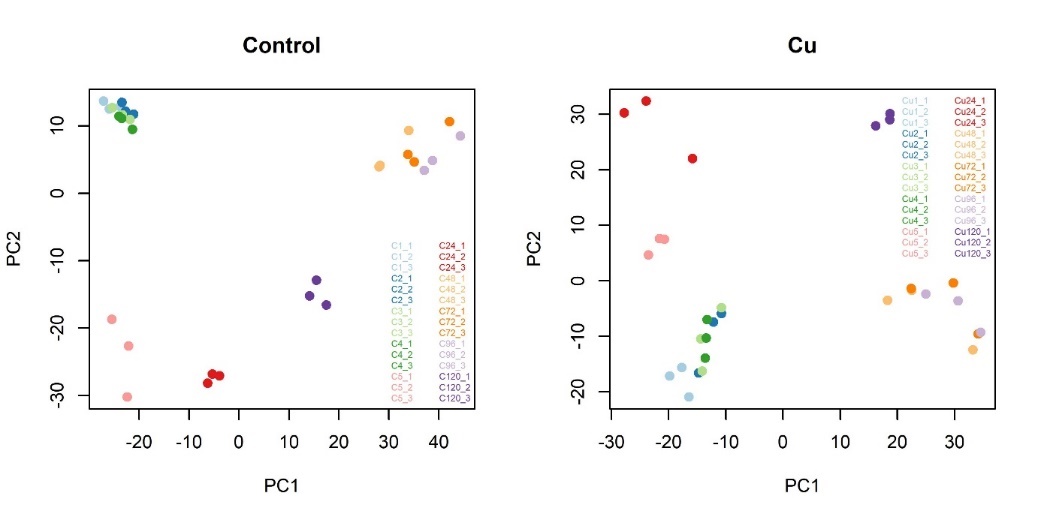


Supplementary Figure S3: PCA diagrams based on Log_2_ normalized intensity values (from protein mass spectrometry) of all replicates from all time points. Left: control condition. Right: Cu condition.


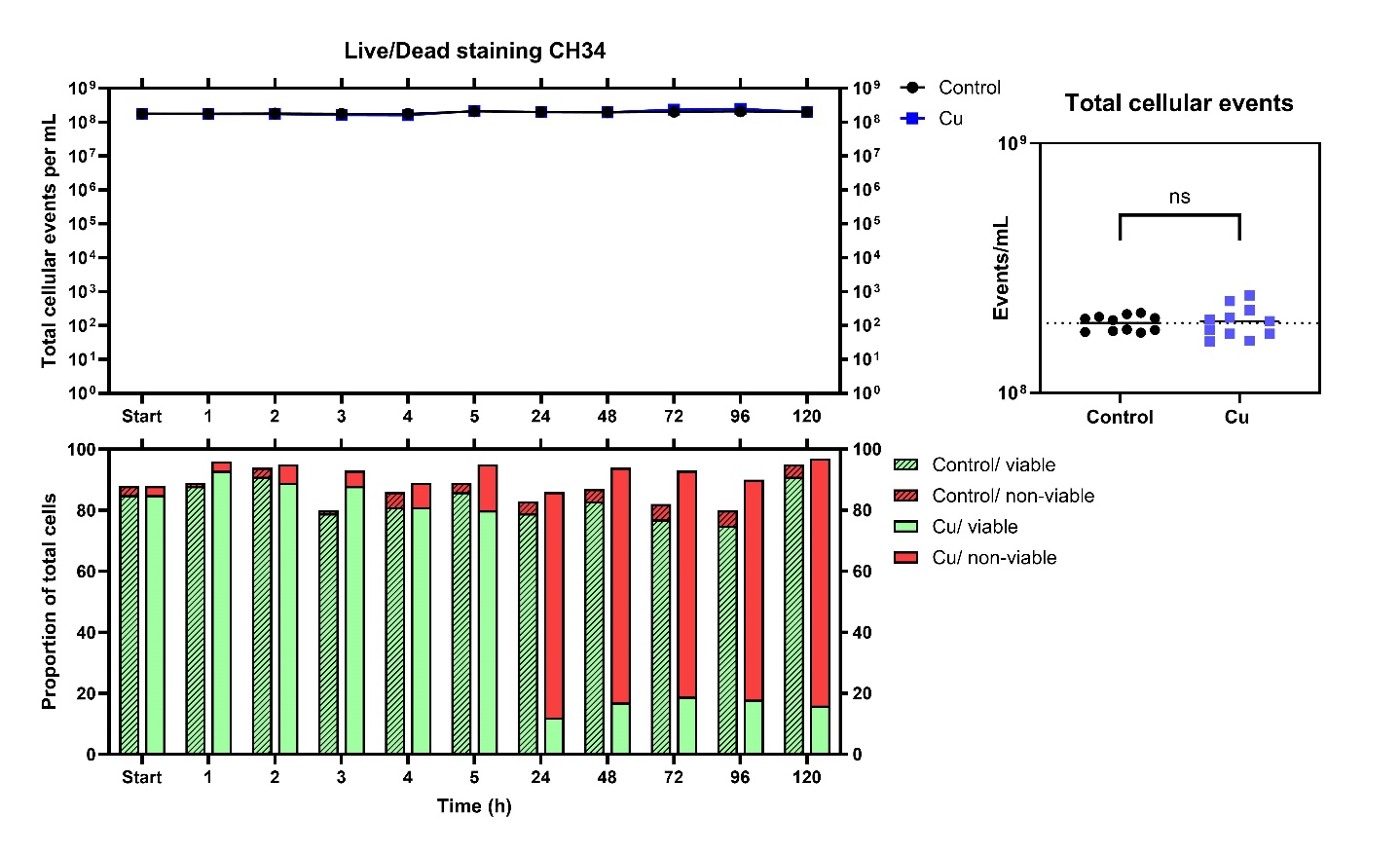


Supplementary Figure S4: Live/dead staining on C. metallidurans CH34 incubated in mineral water ± 10 µM CuSO_4_. Left: Total cell counts in both conditions were constantly > 10^8^ events/mL (which corresponds to the inoculum) over all sample time points and 10-20% of Cu-treated cells remained viable after 24 hours of incubation. Right: Total cell counts based on detected cellular events were not significantly different (ns) between the control and Cu condition, as determined by a Welsh’s t-test.


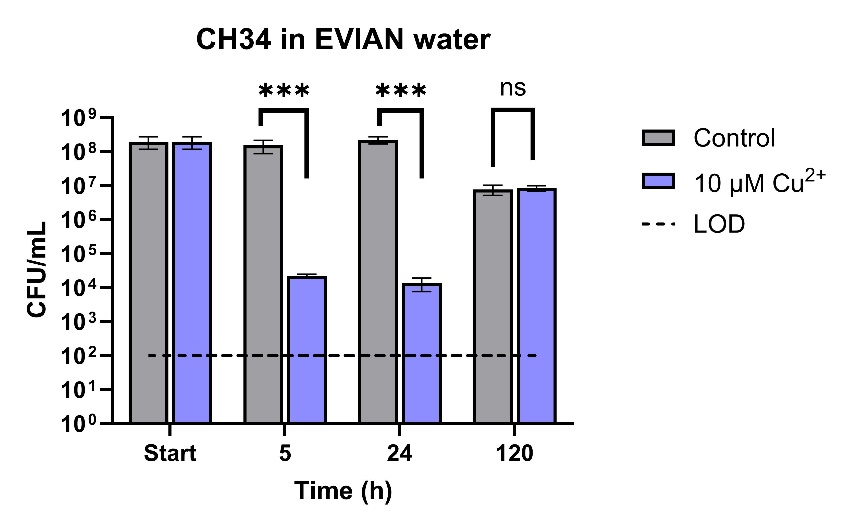


Supplementary Figure S5: The reproducibility of VBNC formation and resuscitation of CH34 was verified by using a different mineral water source (EVIAN). Addition of 10 µM CuSO_4_ caused a log4 reduction of culturability within 5 hours, and cells were completely resuscitated at 120 hours. Significant difference: *** p < 0.001, ns = no significant difference. LOD = limit of detection.


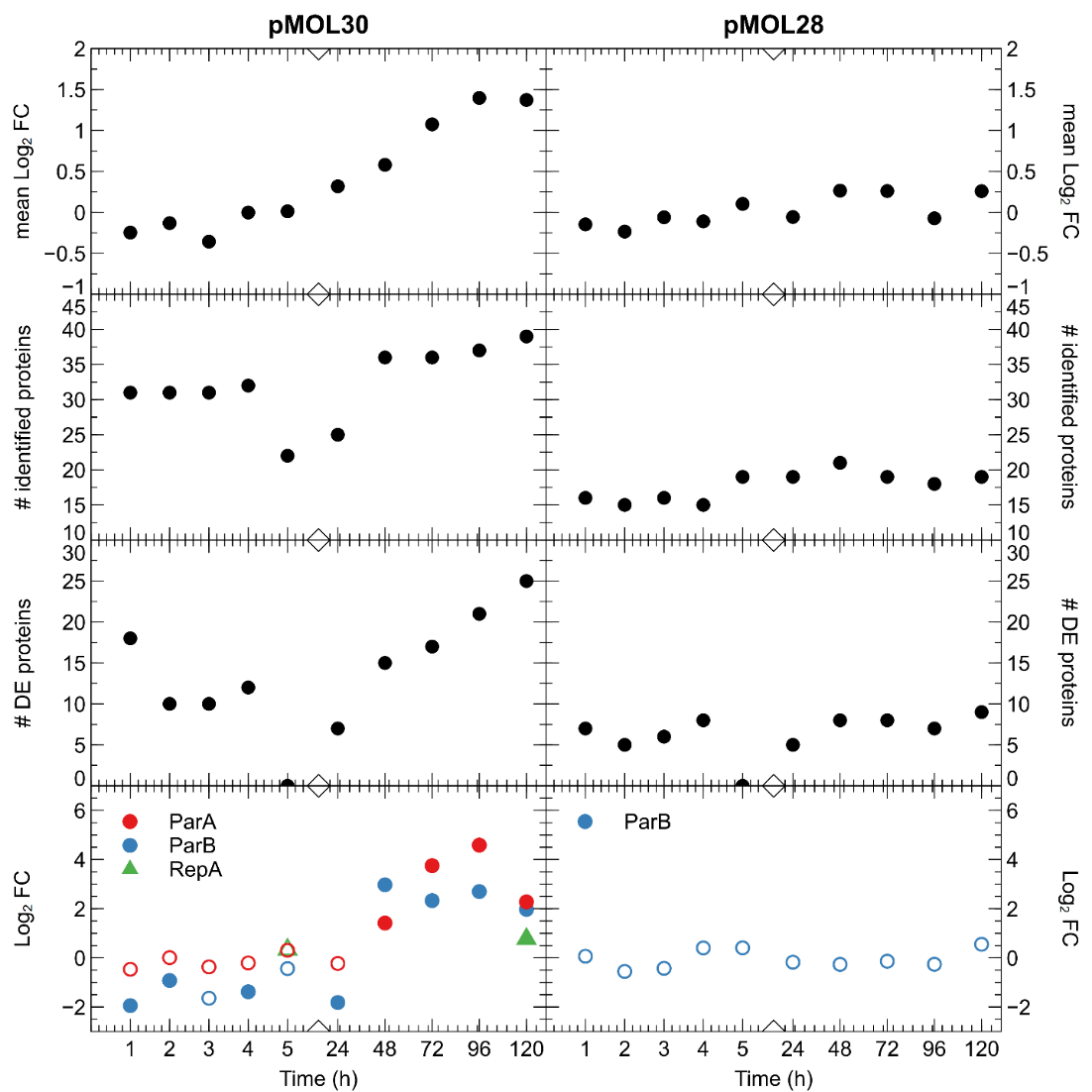


Supplementary Figure S6: The average Log_2_-FC, the number of identified as well as differentially expressed proteins encoded by megaplasmid pMOL30 (left) are gradually increasing during resuscitation, which is not observed for pMOL28 (right). The ParA and ParB partitioning proteins encoded by pMOL30 were upregulated from day 2 onwards, and its replication protein RepA at day 5, which was not observed for pMOL28.


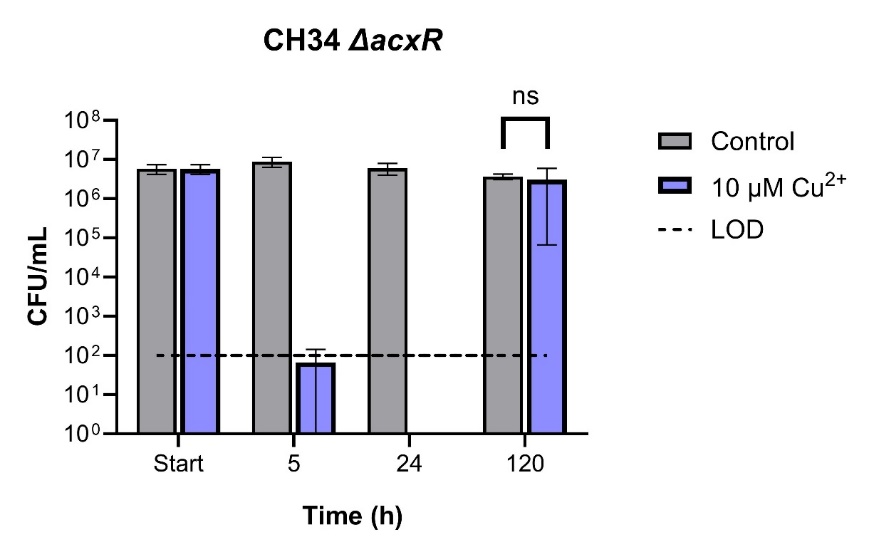


Supplementary Figure S7: The ability to resuscitate in Cu-supplemented mineral water is not affected (120 h) in *C. metallidurans* CH34 when *acxR* was deleted, even though a higher proportion of cells is entering the VBNC state (5-24 h). ns = no significant difference. LOD = limit of detection.


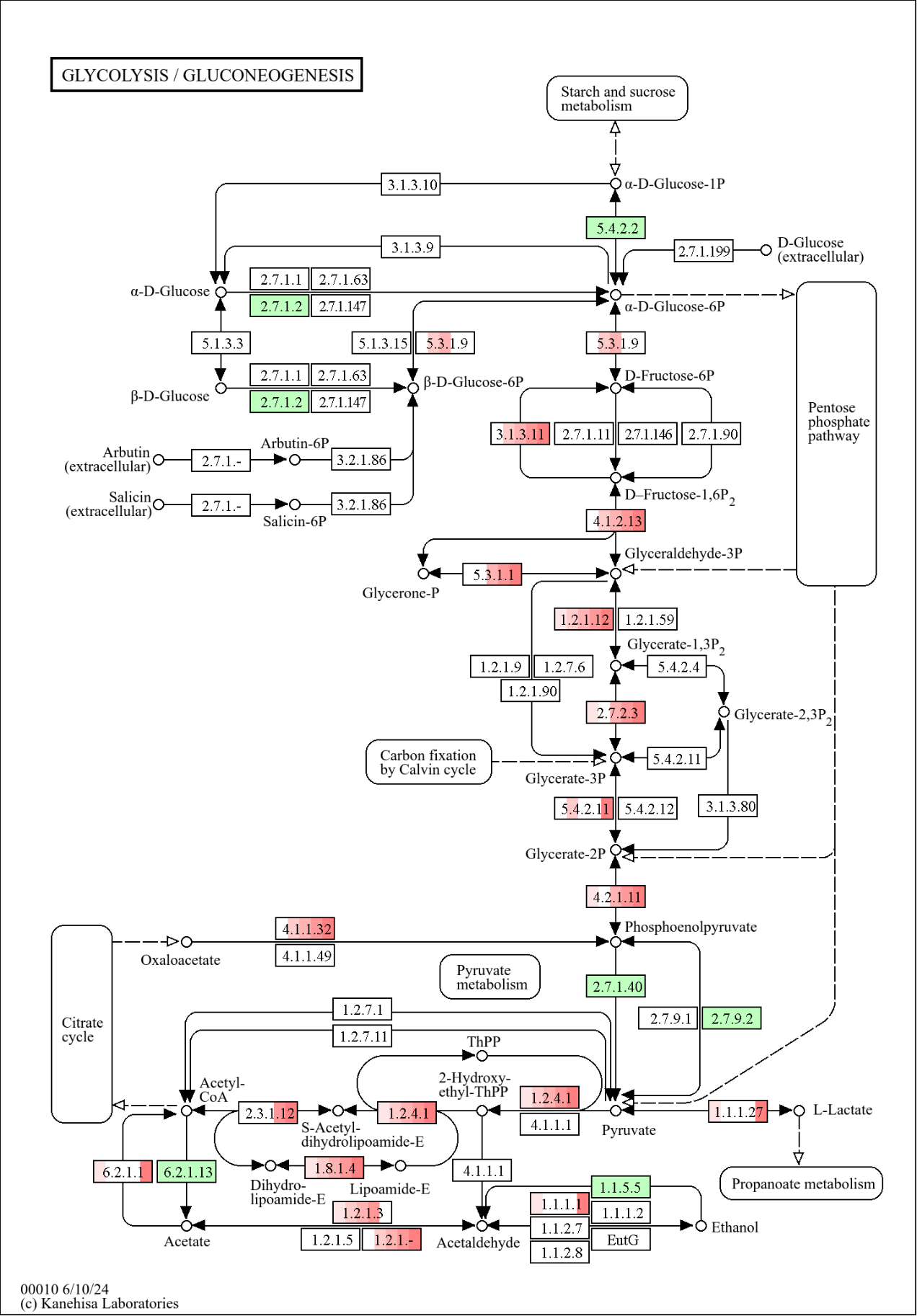


Supplementary Figure S8: KEGG pathway Glycolysis / Gluconeogenesis [PATH:rme00010] in C. metallidurans CH34. Extracted from <https://www.kegg.jp/pathway/rme00010>. Green = present in CH34, non-differentially expressed (Cu vs control condition). Red = differentially expressed at 1, 2, 3, 4 and/or 5 days. White = not identified in CH34.


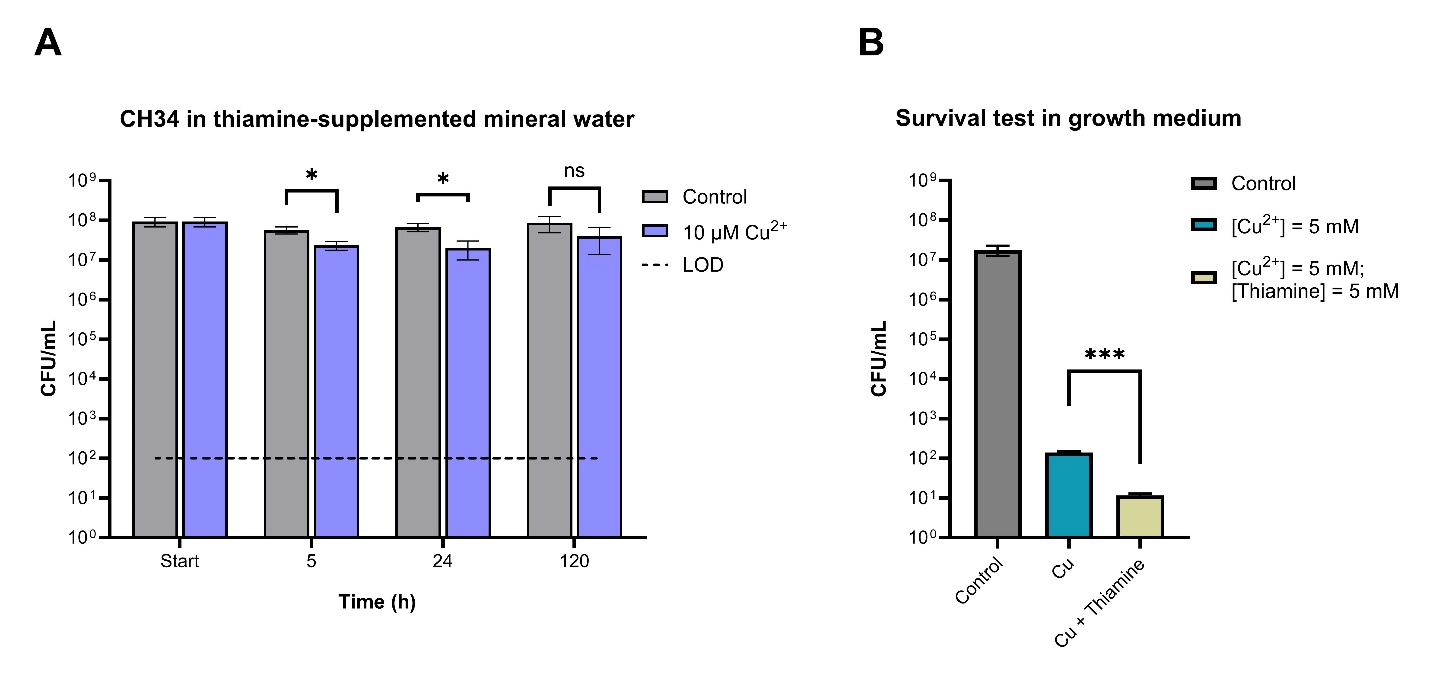


Supplementary Figure S9: **A)** Supplementation of 5.6 mM thiamine to the medium (ORDAL mineral water) impeded loss of culturability when C. metallidurans CH34 was exposed to ± 10 µM CuSO_4_. * significant (p < 0.05) difference, ns = no significant difference. LOD = limit of detection. **B)** The toxic effect of 5 mM Cu^2+^ on CH34 in MM284 (2 hours of exposure) was enhanced by adding 5 mM Thiamine (*** p < 0.001).
